# Supramolecular Gels with Potential Applications as Anti-Icing Agents

**DOI:** 10.1021/acs.langmuir.5c00755

**Published:** 2025-05-23

**Authors:** Nicole K. McLeod, Lee Stokes, Jerry Lewis, David K. Smith

**Affiliations:** † Department of Chemistry, 8748 University of York, Heslington, York YO10 5DD, U.K.; ‡ Kilfrost Ltd, Albion Works, Haltwhistle NE49 0HJ, U.K.

## Abstract

Supramolecular gels based on 1,3:2,4-dibenzylidenesorbitol (DBS) with modifications in the *para* positions of the aromatic rings form effective thickeners for mixtures of monopropylene glycol (MPG) and water, with potential applications as anti-icing fluids. A range of DBS derivatives were tested, and optimal performance was observed for DBS, DBS-OCH_3_, and DBS-SCH_3_. Notably, DBS-SCH_3_ formed gels at concentrations nearly 10-fold lower (<0.1 wt %/vol) than DBS or DBS-OCH_3_, which may be of use in a range of applications. As the amount of water added to MPG increased, gelation ability, gel thermal stability, and rheological stiffness improved as gelator solubility decreased and a solvophobically driven ‘solid-like’ gel network was more easily formed. However, once the water content reached a critical level, gelator solubility became too low and gelation was prevented. DBS-OCH_3_ could tolerate more water than DBS, owing to its higher polarity. The gelators assembled into networks composed of fibers ca. 5–10 nm in diameter. On thermodynamically controlled slow cooling, DBS-SCH_3_ formed a microcrystalline tape-like morphology, but on faster kinetically controlled cooling, more typical of the proposed application, DBS-SCH_3_ assembled into the preferred nanoscale fibrillar network. The gelators were tested in a commercially available aviation deicing fluid (DF+). Each gelator extended the performance of the deicing fluid in a water spray endurance test and, in some cases, provided ‘holdover times’ expected for a higher performance anti-icing fluid. Performance was dependent on gelator loading and the dilution of the DF+ fluid—importantly, holdover times increased with dilution as gel assembly was promoted, indicating that DBS additives may allow the typical amounts of MPG used in such fluids to be lowered. Levels of strain typical of those experienced on aircraft takeoff caused breakdown of the gel as desired for the target application. These LMWGs, therefore, significantly improve the performance of deicing fluids and may be useful additives in the formulation of next-generation anti-icing systems.

## Graphical abstract

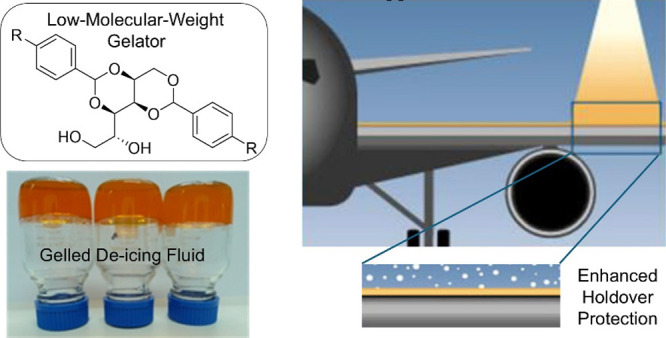

## Introduction

Supramolecular gels form when low-molecular-weight gelators (LMWGs) self-assemble into nanofibers—typically <1 wt %/vol of LMWG is able to immobilize >99% of solvent to create a gel.^1–3^ In general, gels based on polymers dominate industrial applications,^4^ but there are also real-world applications of LMWGs.^5^ For example, the viscosity modification caused by LMWGs has been applied for many years in the lubrication industry.^6^ Supramolecular gels have been applied as deodorant gel sticks in personal care products,^7^ and as glue sticks for adhesive applications.^8^ In polymer technology, self-assembled LMWG networks assist polymer crystallization from the melt phase to fabricate transparent plastics.^9,10^ Optimisation of LMWG structure then allowed development of additives suitable for use in food industry plastics.^11,12^ LMWGs have also been combined with photopolymerisable systems in dental implant technology to limit shrinkage of the polymer resin,^13,14^ and in phase-change inks and 3D printing systems.^15^ In addition to rheological applications of LMWGs, there is burgeoning interest in high-tech applications, like nanoscale electronics or regenerative medicine, where synthetic tunability allows additional function to be programmed into the gel.^5^

An interesting application of modified fluids is in anti-icing—of great importance in a variety of settings, for example the aviation industry.^16,17^ For planes to fly safely, it is essential for all surfaces to be free of ice prior to takeoff—failure can have fatal consequences.^18^ Products are typically glycol-based systems, thickened with polymeric additives.^19^ There are four different types of deicing and anti-icing agent (Types I–IV), which are sprayed onto aircraft in liquid form. Type I agents are deicing agents with high glycol content and low viscosity; they remove frozen deposits from aircraft surfaces. Type II products prevent the buildup of ice (anti-icing) and contain a minimum glycol content of 50% and a pseudoplastic thickening polymer, creating a film on the surface of the aircraft and providing ‘holdover protection’. On takeoff, the shear forces must remove the thickened film. Type III fluids typically have lower holdover times and were designed for use on aircraft with lower takeoff speeds. Type IV products use different thickening agents that significantly extend holdover times.

Beyond aviation, there is widespread interest in anti-icing technology, and in recent years, attention has started to focus on gels in this regard.^20^ However, as yet, it is generally well-established and widely investigated polymer gel systems that are exploited as next generation anti-icing agents.^21–27^ Although the anti-icing industry is dominated by polymer technology, LMWGs could potentially be useful as additives or even replacements. Indeed, there has been emerging interest in LMWGs as cryopreservants, for protecting cells from the adverse impacts of ice by using their self-assembled networks to inhibit its crystallization.^28–30^ The requirements of an industrial anti-icing agent are well-suited to the properties of a LMWG. The system must dissolve in aqueous glycol for spraying (either at ambient temperature or on heating), and when cooled on contact with the freezing surfaces, must form a viscous film that resists the buildup of further ice (Figure 1). This film must be sensitive to shear forces on takeoff. The thermally induced nature of many supramolecular gels,^31^ combined with their shear-sensitive performance,^32^ and the ability of the molecular structure to be tuned to optimize performance in specific solvents,^33^ gives them great potential. Furthermore, in real-world use, anti-icing solutions are often stored at elevated temperatures (ca. 80 °C) for significant periods of time—small molecule LMWGs will typically be more stable under these conditions than polymeric additives. Finally, de/anti-icing produces significant amounts of runoff waste—LMWGs can be selected to be environmentally friendly, and there is also potential to optimize the solvent, minimizing the glycol loading while retaining performance. Although there have been sporadic reports of supramolecular gels that assemble in water/glycol mixtures,^34–36^ there have been no significant attempts to optimize LMWGs for this mixed solvent medium, or to explore anti-icing performance. In this paper, we explore a family of gelators based on 1,3:2,4-dibenzylidenesorbitol (DBS)^37^ for their ability to function in deicing/anti-icing technology (Figure 1). As such, this work yields new insight into supramolecular gels, as well as potentially highlighting a new application for LMWG technology.

**Figure 1:**
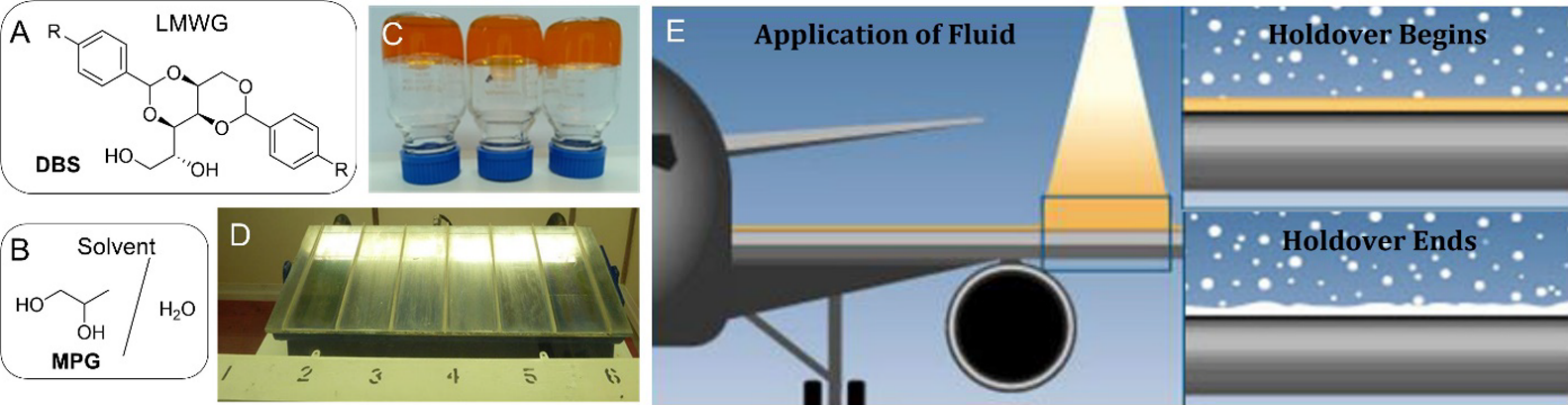
(A) Structure of low-molecular-weight gelator (LMWG) based on 1,3:2,4-dibenzylidenesorbitol (DBS). (B) Structure of solvent used in deicing agents based on mixtures of monopropylene glycol (MPG) and water. (C) Inverted bulk gel samples made in commercial ‘DF+’ at various dilutions of water using DBS (1.0 wt %/vol). (D) Frosticator plate used in the water spray environmental testing (WSET) methodology as a model of the aircraft wing to determine holdover times as ice freezes down the plate. (E) Schematic image of the application of deicing fluid to an aircraft wing and of the holdover effect which ends after a defined time once the aircraft surface is no longer free of ice.

## Experimental Section

### General Experimental Methods

All chemicals and solvents used were commercially available from Sigma-Aldrich or Alfa Aesar and were used without further purification. 1,2-Monopropylene glycol (MPG) was supplied by Kilfrost Limited. The commercial deicing product “Type I DF Plus (DF+)” was provided by Kilfrost Limited and used as supplied. Dibenzylidene-d-sorbitol was purchased from Rika International, commercial name “Geniset D” and was used without further purification. DBS-CONHNH_2_ was prepared through simple two-step synthesis via DBS-CO_2_Me, as described in our previous reports, with characterization data in agreement with that previously published.^38^

For compound characterization, NMR was performed using a JEOL ECX400 spectrometer (^1^H 400 MHz, ^13^C 100 MHz). All chemical shifts (δ) are reported in ppm and referenced to a residual solvent peak. Coupling constant (*J*) values are reported in Hz. ^1^H and ^13^C were assigned with the help of COSY and HSQC spectra. The peaks are reported using the following notation: s—singlet, d—doublet, t—triplet, q—quartet, qn—quintet, m—multiplet. ATR-FTIR was carried out on a PerkinElmer Spectrum 2 fitted with an ATR sampling accessory and Spectrum 10 software. Absorbance bands are reported as wavenumber of maximum absorbance (cm^–1^). Electrospray ionization mass spectroscopy (ESI-MS) was carried out on a Bruker MicroTOF mass spectrometer. Melting points were measured on a Stuart SMP3 using glass capillary tubes. Melting points are recorded as ranges and are uncorrected.

For gel characterization, *T*_gel_ measurements were determined using a thermoregulated oil bath at 1 °C increments. Samples for scanning electron microscopy (SEM) were prepared by spreading a small amount of gel over an aluminum stub and dried in a desiccator. They were then sputter-coated with a 4 nm layer of Au/Pd using a Polaron Agar High Resolution Sputter Coater and imaged with a JEOL JSM-7600F FEGSEM. Transmission electron microscopy (TEM) was performed on copper-backed TEM grids, with samples left to air-dry overnight and imaged using a FEI Tecnai G2 fitted with a CCD camera. Dynamic rheological measurements were performed on a Malvern Kinexus Pro+ rheometer using 20 mm parallel plate geometry and a 1 mm gap. All measurements were performed within the linear viscoelastic region (LVR) and data interpreted using rSpace for Kinexus software. The Water Spray Endurance Test (WSET) was carried out in a climatic (temperature and humidity) controlled tunnel at Kilfrost Limited. All tests were conducted under industry standard conditions at −5 °C.

### General Synthesis of Dibenzylidene-d-Sorbitol-Based Gelators

LMWGs were synthesized via acetal condensation between d-sorbitol and two equivalents of an appropriate aldehyde (Scheme 1). d-Sorbitol (4.90 g, 0.03 mol) was added to a three-necked round-bottom flask fitted with a Dean-Stark trap. A mixture of cyclohexane (35 mL) and methanol (10 mL) was added to the flask. The mixture was stirred and heated to 50 °C for 20 min under nitrogen. In a round-bottom flask the appropriate *para*-substituted benzaldehyde (0.05 mol) was added with *p*-toluene sulfonic acid monohydrate (TsOH) (1.00 g, 5.26 mmol) in methanol (20 mL) which was stirred at room temperature for 20 min. This was then added to the d-sorbitol solution dropwise and the temperature increased to 70 °C for 2–4 h. The mixture was allowed to cool to room temperature where a paste was formed. This was then washed with cold ethanol (3 × 100 mL) to remove any starting materials. The crude product was then dried on the high vacuum line for 2 h and air-dried overnight. Most of the impurities (mono and trisubstituted derivatives) were removed by washing with boiling water (4 × 100 mL) and boiling dichloromethane (4 × 100 mL). The product was finally dried in the vacuum oven at 70 °C overnight. All products were purified to 95%+ purity in-line with industry standards—detailed characterization is in the Supporting Information.

**Scheme 1:**
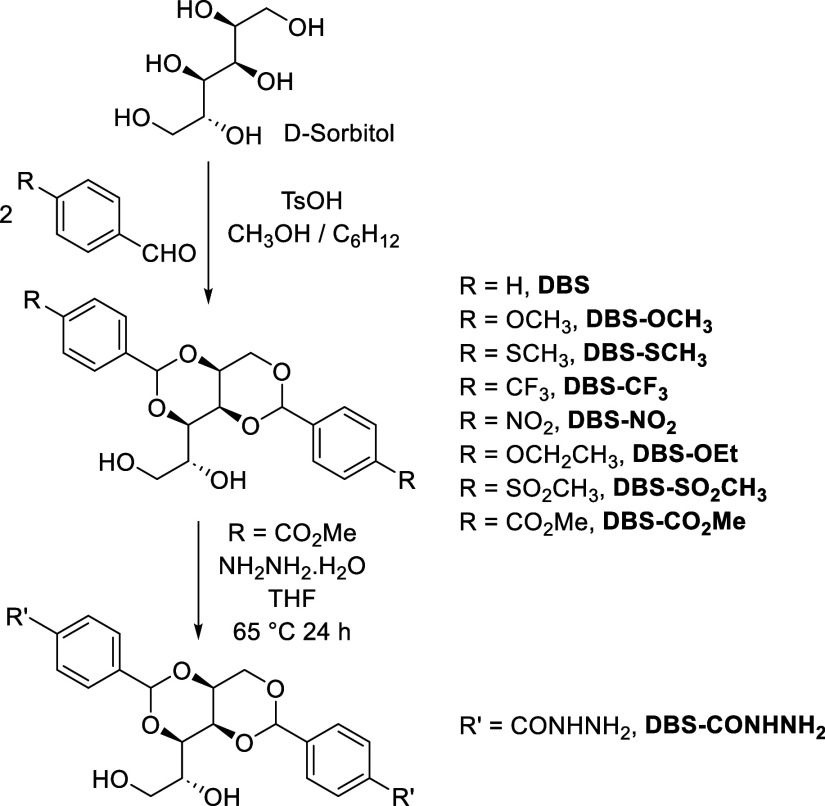
Synthesis of DBS Derivatives and Structures of LMWGs Investigated in This Paper as Gelation Systems in Mixtures of Monopropylene Glycol (MPG) and Water

### Preparation and Preliminary Characterization of Gels

#### Preparation of Gel Samples in MPG:H_2_O

A known amount of gelator was accurately weighed into a 2 mL glass vial. An appropriate mixture of MPG and water (Figure 1B, 1 mL) was then added to the vial using a Gilson pipette. The sample was sonicated for 1 h before being heated in an oil bath to just below the boiling point of the solvent, until a homogeneous solution was formed. The sample was then left at room temperature overnight to cool, over which time the sample forms a gel.

#### Preparation of Gel Samples Using Kilfrost DF+

To prepare gels in deicing fluid, samples were made at 1.0, 0.5 and 0.1% w/v for screening purposes. To make the samples, the solid gelator was accurately weighed out into a 2 mL vial. Solvent based on DF+ at different dilutions (1 mL total volume) was then added to the vial using a Gilson pipet in aliquots as shown in Table S1. Each sample was sonicated for 1 h before being heated in an oil bath to just below the boiling point of the solvent, until a clear homogeneous solution was formed. If, after 1 h of heating, the sample did not form a clear homogeneous solution it was removed from the heat. The samples were left on the bench at room temperature overnight, during which time they formed gels.

#### Determining Minimum Gelation Concentrations (MGCs)

Gels of decreasing concentrations were prepared using the standard method in the sections above. They were then inverted to identify if the gel could support itself under gravity. The lowest concentration at which a gel was stable when inverted was taken as the MGC.

#### Measuring *T*_gel_ Values

Gel samples were used to measure the *T*_gel_ value. The samples were placed in a thermostatic oil bath and the temperature increased at a rate of 1.0 °C min^–1^. As the temperature increased, the gel was removed from the oil bath at every 1 °C increase and inverted (tube inversion test). The temperature at which the gel could no longer support itself under gravity and collapses to the bottom of the vial was recorded as the *T*_gel_, the temperature of the gel–sol transition. All *T*_gel_ values were repeated at least once and averaged.

#### Scaling Up Gel Samples in Kilfrost DF+

Samples were scaled up to 200 mL in glass Schott bottles (Figure 1C). A known amount of gelator was weighed out into a Schott bottle, and solvent (Type I Deicing fluid and water) added as described in Table S2. The samples were sonicated for 1 h then heated in an oil bath to just below the boiling point of water until a clear homogeneous solution was formed. Samples were left overnight for the gels to form and results recorded the next day.

### Rheology

#### Preparation of Rheology Samples

For each sample a known amount of gelator was weighed out into a glass vial (10 mL) and solvent (8 mL of MPG or deicing product) added. The samples were sonicated for 1 h and then heated in an oil bath to just below the boiling point of the solvent until a clear homogeneous solution was formed. The samples were left overnight to form gels at room temperature for testing the next day. As gels can be fragile if applied as a solid, the samples were applied as a solution and the gel allowed to set on the sample plate. The samples were temperature dependent therefore each vial was placed in a water bath and heated to just above the *T*_gel_ value until the solution formed. Using a spoon to reduce any shear or damage to the sample, ∼2 mL of the solution was placed onto the sample stage on the rheometer set to 20 °C and the geometry configured to a gap of 1 mm. The sample was trimmed to remove any excess sample and a hood placed on to the geometry to reduce evaporation before a test was started.

#### Amplitude Sweep

A sample was applied as above and left to equilibrate at 20 °C for 15 min to allow the sample to reach temperature and the gel to form. The sample was then tested across a range of increasing strain (0.001–100%) at a set frequency of 1 Hz and set temperature of 20 °C. This test determines the LVR (linear viscoelastic region) of each sample. A value from the LVR was then used for further tests.

#### Frequency Sweep

The sample was applied as above and left to equilibrate for 10 min on the instrument. Using a known strain value from the LVR the sample was tested across a range of frequencies (0.01–100 Hz) at a set temperature of 20 °C.

#### Variable Temperature Rheology

The samples were applied to the rheometer geometry as described above but this time to a hot sample stage set to 85 °C. The test was carried out using a known strain value within the LVR at a set frequency of 1 Hz on cooling across a temperature range (85 to −5 °C) at increments of −2 °C/min. The test was then equilibrated for 5 min at −5 °C before completing a heating cycle at +2 °C/min from −5 to 100 °C. Temperature ramps provide information on the behavior of gel samples to both increasing and decreasing temperature and allow determination of *T*_sol–gel_ (either as the point at which gel assembly was complete, *T*_f_, or the onset of gel formation, onset-*T*_f_) and *T*_gel–sol_ (complete gel dissolution, *T*_d_).

#### Time-Resolved Rheology

Each sample was applied hot to a 20 °C sample stage and the test started as quickly as possible. The test uses a known strain within the LVR at a set frequency of 1 Hz at a set temperature, 20 °C, over 1 h to characterize how fast a gel can form under given fixed conditions.

### Scanning Electron Microscopy

Samples for SEM imaging were prepared in two ways (see below). Once the samples were prepared and the xerogels formed, the samples were coated with a 4 nm layer of Au/Pd using a Polaron High Resolution Sputter coater and were imaged using a JEOL 7600F FEG-SEM.

#### Slow Cooling Conditions

Gels were formed as described above. Once the gels had set, a small amount of gel was removed with a spatula and spread thinly onto an aluminum SEM stub. These were then placed on a polystyrene holder and placed in a desiccator to air-dry for 2 days to 2 weeks depending on the solvent, to leave the xerogel.

#### Fast Cooling Conditions

Gels were formed as described above. They were then placed into a hot oil bath at 98 °C to fully dissolve. During this time the SEM stubs on a polystyrene holder were placed in the freezer at −21 °C for 5 min to reach low temperature. One drop of the hot solution was then placed on to the cold SEM stub in the freezer, using a Pasteur pipet and spread over the surface. The stubs were placed back into the freezer for 2 h to form gels at low temperatures. After 2 h, the stubs were then placed in a desiccator at room temperature for the sample to dry (3 days), leaving the xerogel.

### Water Spray Endurance Test (WSET)

The water spray endurance test is a laboratory-based test developed to evaluate the holdover performance of deicing and anti-icing fluids under freezing conditions.^39,40^ This test determines the length of time an aircraft has between application of a de/anti-icing fluid to taxiing and takeoff before reapplication is required due to further ice contamination. This test was carried out within a temperature-controlled climatic chamber, and conducted at −5 °C. Within this chamber, an aluminum frosticator plate (Figure 1D), representative of an aircraft’s leading edge on a wing was setup at −5 °C with a 10° angle. The frosticator consists of 6 test panels. Four panels were used for test samples with the remaining two acting as controls, with the use of aluminum square plates which are weighed before and after the test to identify the weight of ice formed throughout the test known as the “catch”. The weight of the ice (“catch”) formed during the test was ∼5 ± 0.2 g/dm^2^ h^–1^ as detailed in AS5901.

Each sample (75 mL) was applied to the top of the aluminum test panels by pouring the fluid from left to right ensuring the top lip (leading edge) was covered. The sample flowed down the test panel through gravitational forces and wet the panel. This was repeated for every sample tested. Once all samples were applied, they were then left for 5 min on the frosticator plate to reach temperature. After 5 min, the test was started by turning on the motor above the frosticator plate which holds a spray nozzle. This nozzle sprays water at a rate of 0.5 mm creating a fine mist which moves forward and backward over the frosticator plate covering the samples. As soon as the spray started, the time was recorded. Gravity causes the fluid to run down the plate and coat it, however, this makes the fluid thinner at the top compared to the bottom. As the test progresses frost forms from the top edge of the panels and works its way downward. At 25 mm from the top edge is a line indicating the end of the test zone. As soon as the first shard of ice touched this line, the time was recorded and used to calculate the holdover time for each sample. Each sample was treated in this way and when all samples were complete the test was stopped and the end time recorded. The control plates were weighed after the test and used to calculate the catch. Using the catch, end time of experiment, and the completion time for each sample, holdover values were calculated. All WSET tests were carried out twice and the results averaged.

Samples tested using the WSET are usually solutions at room temperature, but the gelator samples in Type I DF+ were gels at room temperature and hence required a different approach. All deicing samples prepared as described above with gelator incorporated into them were heated in an oil bath at 85 °C until they formed solutions. A 75 mL aliquot was then transferred to a beaker, the sample applied immediately to the frosticator plate while hot, and the test conducted as described above. Control samples of the original Type I DF+ were diluted in the same way as the gel samples. These were then tested via WSET and used for comparison with Type I DF+ samples with added gelator.

### Aerodynamic Testing Using Rheology

To determine the effects of shear on gel samples and to identify if these gels would break down and be able to be removed from an aircraft after takeoff, variable shear rheological analysis was performed. Samples were applied hot to the rheometer stage as described previously, with the stage set at 20 °C. Each sample was set to equilibrate for 15 min before the test was started, to allow the gel to form. This test was then set to use a strain within the LVR with a frequency of 1 Hz at 20 °C for 10 min. The parameters were then changed to apply a strain of 10% (outside of the LVR) for 10 min with the remaining parameters staying constant. Finally, the strain was returned to the initial value for 1 h. These steps were run consecutively with no breaks in between. This characterizes the ability of the gel to set up on a surface, be broken down and then finally recover (‘self-heal’). To further understand the effects of increasing strain, the test was repeated twice more for each sample applying instead 50 or 100% strain in the second step of the experiment.

## Results and Discussion

### Choice of Gelators

We decided to focus on developing LMWGs with low-cost, high commercial relevance, and versatility in terms of solvent compatibility. We selected 1,3:2,4-dibenzylidenesorbitol (DBS) as a potential candidate—this low-cost LMWG, based on the condensation between sorbitol and two equivalents of benzaldehyde (Scheme 1) has been known for >125 years, can be synthesized on large scale, and is well-established in a range of high-volume industrial settings^37^ DBS is a ‘butterfly surfactant’ that self-assembles as a result of well-defined hydrogen bond interactions between the sorbitol ‘bodies’, and the solvophobic effect/stacking of the aromatic ‘wings’.^41^ The balance of these interactions depends on the solvent,^42–44^ meaning this gelator has broad scope and is capable of immobilizing a wide range of organic solvents.^45–48^ This stacking process leads to a one-dimensional columnar assembly of DBS molecules into fibrils, then then bundle further to give rise to the ca. 10 nm flexible nanofibers that typically underpin gels form by this type of gelator. Furthermore, DBS has great potential for synthetic variation as different aldehydes can easily be used in its synthesis, thus incorporating different functional groups on the ‘wing-tips’, hence mediating interactions between the self-assembled gelator and the solvent phase—in this way, a variety of organogelators have been reported.^49–51^ We previously developed a family of DBS hydrogelators (e.g., DBS-CONHNH_2_), extending the solvent range of DBS out of organic solvents and into water.^38,52^

To further support our choice of DBS-based gels for this application, we considered the performance of this LMWG in terms of Hansen solubility parameters (HSPs).^53^ These parameters reflect the characteristics of a substance—specifically representing the contributions of dispersion interactions (δ_d_), dipolar interactions (δ_p_) and hydrogen bonding interactions (δ_h_) to the cohesive energy. The HSPs of different solvents have been shown to have very good predictive capacity for gelation potential of a variety of LMWGs.^33,54^ The behavior of LMWGs can be understood in terms of overlapping spheres of Hansen space that describe different types of behavior. For example, when considering 1–5 wt %/vol DBS in different solvents, it has been shown that the molecule forms solutions within a sphere centered at HSPs of δ_d_ = 18.3 MPa^0.5^, δ_p_ = 14.1 MPa^0.5^, δ_h_ = 9.3 MPa^0.5^ and having a radius (based on a plot of 2δ_d_, δ_p_ and δ_h_) of 9.0 MPa^0.5^.^46^ DBS then forms transparent gels within a larger sphere centered at HSPs of δ_d_ = 16.7 MPa^0.5^, δ_p_ = 8.5 MPa^0.5^, δ_h_ = 22.7 MPa^0.5^ and having a radius of 21.1 MPa^0.5^. This gel sphere has the solution sphere nested within it. There is also a slightly different sphere which describes opaque gels. Outside of the two gel spheres, DBS is insoluble and does not form gels. By comparing the solution and gel spheres with the HSPs of a given solvent, it is possible to predict the way DBS will behave. The standard solvent used for deicing is monopropylene glycol (MPG), which has Hammett parameters: δ_d_ = 16.8 MPa^0.5^, δ_p_ = 9.4 MPa^0.5^, δ_h_ = 23.3 MPa^0.5^. It is possible to calculate the distance of these parameters for a solvent i from the center of each sphere for a substance j as defined by eq 1 and hence determine whether the solvent is within the radius.

$$R_{\mathrm{ij}}=(4(\delta_{\mathrm{di}}-\delta_{\mathrm{dj}}{)}^{2}+(\delta_{\mathrm{pi}}-\delta_{\mathrm{pj}}{)}^{2}+(\delta_{\mathrm{hi}}-\delta_{\mathrm{hj}}{)}^{2}{)}^{1/2}$$ 1

Applying this equation to MPG and considering the center of the ‘solution’ sphere for DBS,^46^ yields a distance of 15.1 MPa^0.5^, outside the ‘solution’ sphere. Calculating the distance of MPG from the center of the gel sphere gives a value of just 1.2 MPa^0.5^. Indeed, MPG sits close to the center of the sphere for gel formation and we therefore proposed it was an ideal candidate for developing deicing solutions in which MPG will be mixed with water, causing the HSPs to be shifted away from the values for pure MPG (see further discussion below). We therefore decided to study the performance of DBS derivatives as LMWGs in solvent systems relevant to anti-icing technology, and determine their potential, as well as probing the impact of synthetic modification.

### Synthesis of Gelators

A family of DBS derivatives was synthesized (Scheme 1) simply by choosing the relevant para-substituted aldehyde and performing an acid-catalyzed condensation reaction of two equivalents of the aldehyde with sorbitol. In each case, the reactions gave rise to the desired DBS derivative as well as small amounts of the monobenzylidenesorbitol (MBS) and tribenzylidenesorbitol (TBS) byproducts. The MBS byproducts were removed by washing with boiling water, and TBS byproducts by washing with dichloromethane. Washing with cold ethanol removed unreacted starting material and catalyst. The desired products were obtained in acceptable to excellent yields (18–88%). This method is based on the well-established literature approach for DBS and its derivatives, indeed some derivatives synthesized here have been reported by ourselves (DBS-CO_2_Me and DBS-CONHNH_2_) and others (DBS-OCH_3_ and DBS-NO_2_) previously.^38,49–51^ Characterization data were in agreement with published data and/or expectations for this class of molecule and are presented in the Supporting Information.

### Gel Formation and Characterization in Mixtures of Monopropylene Glycol (MPG) and Water

We initially tested the ability of these DBS derivatives to form gels in solvents ranging from 100% water to 100% monopropylene glycol (MPG) (Table 1). In general, it was expected that water would enhance the self-assembly of DBS (and derivatives) by amplifying the solvophobic effect which encourages packing of the aromatic rings and solvophobic surfaces into the assembled nanostructure, promoting gelation as the water content increased. However, once too much water is present, then it will not be possible to fully solubilize the DBS (and derivatives) in the first place because the solvophobic interactions between LMWGs are too great, and hence at a certain water content, compounds would be expected to be insoluble, and unable to form gels. As such, water content should play an active and important role in controlling the gel assembly process.

**Table 1:** Loadings at Which Gelation Was Observed for Various DBS Derivatives in Mixtures of Monopropylene Glycol (MPG) and Water^a^

	gelation ability (and range of loadings in % wt/vol)			
gelator	100% MPG	75:25 MPG:H_2_O	50:50 MPG:H_2_O	100% H_2_O
DBS-CONHNH_2_	I	G (0.7–1.0)	G (0.8–1.0)	G (0.2–0.35)
DBS-SO_2_CH_3_	I	G (0.3–1.0)	G (0.2–1.0)	I
DBS-OCH_3_	G (0.2–1.0)	G (0.1–1.0)	G (0.1–1.0)	I
DBS-SCH_3_	G (0.3–0.9)	G (0.03–0.9)	G (0.02–0.4)	I
DBS	S	G (0.1–1.0)	G (0.07–1.0)	I
DBS-CF_3_	S	G (0.1–0.9)	G (0.1–0.9)	I
DBS-NO_2_	S	G (0.2–0.9)	I	I
DBS-OCH_2_CH_3_	S	I	I	I

a The gelators are presented in approximate order from more polar at the top to less polar at the bottom to emphasize trends in gelation ability. I = insoluble, S = soluble, G = gel. Numbers in parentheses represent the range of wt %/vol at which gels were formed.

It was evident that the different derivatives had significantly different gelation potentials, as might be expected based on the differences in solvophobicity induced by the peripheral functional groups—this modifies the solubility of the LMWG and provides a solvophobic driving force for gel assembly. The only true hydrogelator was our previously reported DBS-CONHNH_2_, which has relatively polar substituents and formed gels in the range 0.20–0.35 wt %/vol in 100% water.^38^ The relatively poor ability of DBS and apolar derivatives to form true hydrogels can be understood by considering the HSPs of water: δ_d_ 15.6 MPa^0.5^, δ_p_ 16.0 MPa^0.5^ and δ_h_ 42.3 MPa^0.5^. For unmodified DBS in pure water, the distance between the solvent HSPs and those for the center of the gelation sphere in Hansen space is 21.1 MPa^0.5^, right on the edge of the gelation sphere (radius 21.1 MPa^0.5^). As such, DBS is insufficiently soluble in water to form homogeneous hydrogels–the same is true of many of its derivatives with apolar substituents.

In 100% MPG, however, if the peripheral groups were polar (e.g., DBS-CONHNH_2_, DBS-SO_2_CH_3_), the LMWG was not able to assemble into gels due to lack of solubility. Conversely, if the peripheral groups were too apolar (e.g., DBS-OCH_2_CH_3_, DBS-CF_3_ etc.) then the LMWG tended to be too soluble in 100% MPG to assemble into gels—clearly in terms of HSPs, these derivatives must have a solubility sphere that includes MPG. It might seem surprising that DBS was soluble in 100% MPG given the prediction from HSPs that it should form gels in this solvent (see above). However, it is important to note that Rogers and co-workers’ study of DBS in terms of HSPs considered solubility/gelation criteria at loadings of 1–5 wt %/vol.^46^ In this work on deicing agents, however, we are interested in significantly lower loadings of LMWG (0.01–1.0 wt %/vol) as these have greater commercial relevance and would offer less environmental burden. Obviously, operating at significantly lower loadings will mean LMWGs are more likely to remain soluble, whereas at the higher loadings of previous work,^46^ gelation will be favored. Therefore, in 100% MPG, only DBS-OCH_3_ and DBS-SCH_3_ had the appropriate balance between solubility and insolubility/assembly to form gels at the desired concentration range. Interestingly, even simply changing the peripheral functionalization from methoxy (DBS-OCH_3_) to ethoxy (DBS-OCH_2_CH_3_) effectively switched off the ability to form gels by changing the solubility. This indicates how relatively small changes in molecular structure can have significant impact on gelation ability.

In mixed MPG:H_2_O solvents, typical of deicing and anti-icing agents, many of the derivatives formed effective gels, however, the least polar gelators tended to be too insoluble to fully dissolve on heating. Lack of complete solubility is a particular problem here as not only does it lead to inhomogeneous gels, it is incompatible with spraying a fully dissolved hot deicing solution in the ultimate target application. The precise solvent mixtures that could be gelated correlated with the polarity/solubility of the gelator and its potential to interact through solvophobic assembly. The results suggest that the solubility and assembly potential of this class of molecule is broadly balanced in the right range for gelation in mixed MPG:H_2_O solvent systems, confirming our hypothesis that DBS gelators have significant potential for anti-icing.

Thinking about mixed solvent systems, HSPs can have significant use—indeed, HSPs have previously been used to study the performance of LMWGs in binary solvent systems.^55^ In water, the polar and hydrogen bonding parameters are significantly larger than those for MPG (Δδ_p_ = +6.6 MPa^0.5^, Δδ_h_ = +19.0 MPa^0.5^) while the dispersion parameter is somewhat lower (Δδ_d_ = −1.2 MPa^0.5^). In binary mixtures of MPG and H_2_O, the HSPs will vary from one extreme to the other, and it is usually assumed that this variation is linear (see Figure S6 for HSPs of mixtures of MPG:H_2_O).^53^ Given that the HSPs for MPG are close to the center of the previously defined gelation sphere, while the values for water sit on its periphery, we predicted that gelation should operate (to varying extents) across this range of solvent mixtures. Indeed, considering the data in Table 1, it is clear many DBS derivatives form effective gels in mixed MPG:H_2_O systems. We considered performing a fuller HSP analysis, but this would have required a lot more data than available here, and given our primary goal was to develop novel deicing and anti-icing agents, we were happy to use HSPs as a guiding principle, rather than performing full detailed analysis, especially as this has been done previously for the parent DBS and some derivatives before. Nonetheless, it is evident that substituents will introduce subtle differences in the compatibility of the LMWGs with the solvent, and hence slightly shift the solubility and gelation spheres, as has been previously reported for families of other related LMWGs.^47,56^

The minimum gelation concentrations (MGCs) observed for these compounds were typically ca. 0.1 wt %/vol, i.e., 0.1% of LMWG can effectively immobilize 99.9% of solvent. However, the more polar LMWGs (DBS-CONHNH_2_ and DBS-SO_2_CH_3_) had higher MGCs as their greater solubility gives them less tendency to assemble. Impressively, DBS-SCH_3_ had an MGC value as low as 0.02 wt %/vol in 50:50 MPG:H_2_O. This is a remarkable performance for any LMWG and indicates an order of magnitude difference in gelation ability between DBS-SCH_3_ and other members of this family. It is possible that sulfur–sulfur interactions^57^ or chalcogen bonding^58^ may reinforce this gel, hence lowering the MGC, and/or that increased solvophobicity plays a key role.

We determined the thermal stabilities of these gels by monitoring the temperature at which the gel is converted into a sol (*T*_gel_) using a simple, reproducible tube inversion methodology. Figure 2A-C illustrates the *T*_gel_ values for our preferred gelators DBS, DBS-OCH_3_ and DBS-SCH_3,_ with all gelators showing the expected concentration-dependent increases in *T*_gel_. As noted above, DBS-SCH_3_ forms gels at much lower concentrations than the other LMWGs. Furthermore, in 50:50 MPG:H_2_O (Figure 2A) and 75:25 MPG:H_2_O (Figure 2B), the DBS-SCH_3_ gels have significantly higher thermal stability than either DBS or DBS-OCH_3_. Given the widespread commercial use of DBS in a variety of industrial settings, we suggest that the enhanced performance of DBS-SCH_3_ may give it practical uses in well-established DBS applications in terms of lowering the amount of additive required, potentially by an order of magnitude. This would potentially have high value on both economic and environmental grounds.

**Figure 2:**
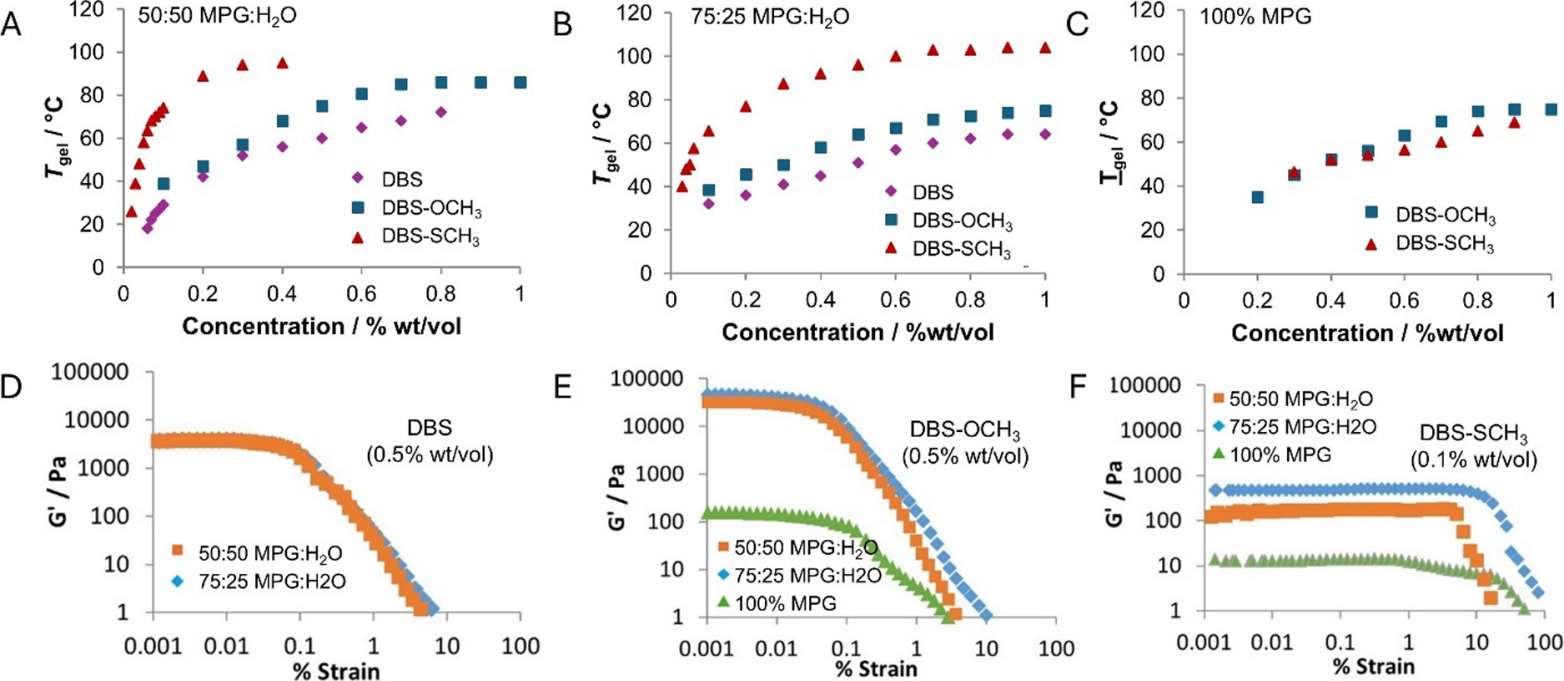
(A–C) Concentration-dependent *T*_gel_ values in (A) 50:50 MPG:H_2_O, (B) 75:25 MPG:H_2_O, and (C) 100% MPG for different LMWGs (purple diamonds = DBS, blue squares = DBS-OCH_3_, red triangles = DBS-SCH_3_). (D–F) *G*′ values of (D) DBS (0.5 wt %/vol), (E) DBS-OCH_3_ (0.5 wt %/vol), and (F) DBS-SCH_3_ (0.1 wt %/vol) in different ratios of MPG:H_2_O (orange squares = 50:50 MPG:H_2_O, blue diamonds = 75:25 MPG:H_2_O; green triangles = 100% MPG).

In 100% MPG, the behavior of DBS-SCH_3_ was much more similar to DBS-OCH_3_ (Figure 2C), while DBS was too soluble to form gels. We suggest the relatively poorer performance of DBS-SCH_3_ in this solvent reflects its increased solubility in the fully organic solvent. Clearly, the presence of some water is highly beneficial to drive the self-assembly of DBS-SCH_3_ compared with DBS-OCH_3_, which would support the view there is a significantly greater degree of hydrophobicity associated with this more polarizable, less hydrogen bonding, thioether. For all three LMWGs, we found that, to some extent, increasing the percentage of water in the gel increased *T*_gel_—consistent with the view that as more water is introduced, the gelator becomes less soluble, and assembly into solid-like fibers is favored. However, if too much water is present (e.g., 100% water), the system becomes too insoluble for gel assembly. The impact of water on gel self-assembly has been the subject of interest, and the results here are aligned with others in the literature.^55,59^

We also explored the thermal stability of some of the other DBS derivatives (Figure S1), although these did not form gels across the full range of solvent mixtures. Focusing on the gels in 75:25 MPG:H_2_O, the more hydrophobic gelators (DBS-CF_3_ and DBS-NO_2_) formed more thermally stable gels than the more hydrophilic gelators (DBS-SO_2_CH_3_ and DBS-CONHNH_2_). This supports the view that LMWG solvophobicity drives self-assembly—the more favored this process, then the more thermally stable the gels become. On moving to a more polar solvent with greater water content (50:50 MPG:H_2_O), the gels largely improve their thermal stability as the hydrophobic effect becomes increasingly dominant. However, if the gelators are too hydrophobic to become soluble, such as DBS-NO_2_ in 50:50 MPG:H_2_O, then they are unable to form gels.

After screening the family of LMWGs in this way, the results confirmed that DBS-OCH_3_ and DBS-SCH_3_ seemed like good lead compounds based on the scope of solvent mixtures they effectively gelled, while DBS was also a good candidate based on its ready availability and approved status in a variety of industrial applications.^37^

We investigated the rheological performance of our preferred gels using a rheometer in oscillatory shear mode with a parallel plate geometry. In the first event, we determined the response of gel elastic modulus (*G*′) to increasing strain. Gels based on DBS were essentially independent of solvent variation (75:25 MPG:H_2_O and 50:50 MPG:H_2_O), with *G*′ values of ca. 3800 Pa and a yield stress of 0.3% strain (Figure 2D). For DBS-OCH_3_, the gel was also very similar in performance in 75:25 MPG:H_2_O and 50:50 MPG:H_2_O, but gel stiffnesses were significantly higher than for DBS, with *G*′ values an order of magnitude higher, at ca. 30000 Pa (Figure 2E), although the yield stress remained low at 0.2% strain. On studying DBS-OCH_3_ in 100% MPG, the *G*′ value dropped to just 130 Pa. This is in-line with a view that once water is absent, it disfavors the hydrophobic assembly mode that otherwise drives the formation of more effective gels with greater stiffnesses.

For DBS-SCH_3_, the *G*′ values were significantly lower—only 160 Pa in 50:50 MPG:H_2_O, dropping to just 13 Pa in 100% MPG (Figure 2F). However, the loading of this gelator was only 0.1 wt %/vol, compared to 0.5 wt %/vol of the other two gelators, reflecting the more effective assembly of DBS-SCH_3_ described above, hence softer gels might be expected. The reason for using this lower concentration is that DBS-SCH_3_ is not fully soluble at higher loadings, leading to nonhomogeneous gels. Meanwhile, the other LMWGs did not form gels at low loadings of 0.1 wt %/vol, and could not therefore be studied under these conditions. Interestingly, DBS-SCH_3_ gels had higher yield stress values of up to ca. 8% for DBS-SCH_3_ in 75:25 MPG:H_2_O. The DBS-SCH_3_ gel networks are therefore softer, but probably as a result, are better able to resist the application of strain without breaking down. Indeed, this inverse correlation between gel stiffness and yield stress is commonly seen in supramolecular gels.^60^

Putting this rheology into the context of our desired application, typical anti-icing fluids have low *G*′ values of only 1–10 Pa. As such, most of the gels produced here are significantly stiffer, giving them potential to establish thicker films, which could provide long-term protection. Typical anti-icing fluids have strain resistances of 1–10%; but for these supramolecular gels, we mostly observe lower values. This would be an advantage in aviation applications as the removal of the gel on takeoff is of key importance. Stiffer gels such as these, which could be completely removed on takeoff, could potentially offer a step-change in anti-icing technology.

Frequency scans of the gels were performed. DBS and DBS-OCH_3_ had *G*′ values that were an order of magnitude larger than *G*″, indicative of gel-phase behavior. The performance was independent of frequency from 0.1 to 100 Hz (Figure S7). For DBS-SCH_3_, *G*′ was independent of frequency up to ca. 10 Hz, probably reflecting the lower loading of the LMWG (Figure S8).

We then carried out temperature-dependent rheology, cooling the gels from ca. 80 to −5 °C on the rheometer plate and then reheating (Figure 2). This technique allows clear visualization of the onset of gel behavior on cooling as G* rapidly increases, and its loss on heating as G* decreases again. It also provides insight into the hysteresis between heating and cooling. We define the temperature of gel formation *T*_f_ as the temperature at which G* reaches its maximum value, and the temperature of gel disassembly *T*_d_ as the temperature at which G* reaches its minimum value again (Table 2).

**Table 2:** Temperatures for Gel Formation on Cooling (*T*_f_/°C) and Gel Breakdown on Heating (*T*_d_/°C) as Determined via Variable Temperature Rheology for DBS (0.5 wt %/vol), DBS-OCH_3_ (0.5 wt %/vol), and DBS-SCH_3_ (0.1 wt %/vol)^a^

		solvent		
gelator		100% MPG	75:25 MPG:H_2_O	50:50 MPG:H_2_O
DBS (0.5 wt %/vol)	*T*_f_/°C	no gel	18.7	19.8
	*T*_d_/°C	no gel	76.4	89.7
DBS-OCH_3_ (0.5 wt %/vol)	*T*_f_/°C	18.8	35.7	45.5
	*T*_d_/°C	83.8	95.6	96.9
DBS-SCH_3_ (0.1 wt %/vol)	*T*_f_/°C	0.3	6.2	20.9
	*T*_d_/°C	63.2	70.8	84.8

a *N* = 1, errors ± 1.0 °C.

As expected, on increasing the water content of the solvent (left to right in Table 2), all of the phase transition temperatures increased, as hydrophobically driven gel assembly is enhanced, in agreement with the *T*_gel_ values described above (Figure 2A–C). In 75:25 MPG:H_2_O, DBS fully assembles into a gel on cooling to ca. 20 °C (Figure 3), while for DBS-OCH_3_, this occurs at ca. 35 °C, indicating the more favored assembly of this latter gelator (it also formed the stiffer networks, Figure 2D). Similar trends were followed in the solvents with greater MPG content. For DBS-SCH_3_, assembly into a gel occurred at ca. 21 °C in 50:50 MPG:H_2_O, but this gelator is being tested at significantly lower loading (0.1 wt %/vol) so the lower transition temperature than DBS-OCH_3_ is not surprising—indeed, it was impressive that DBS-SCH_3_ could match the thermal performance of DBS, which was being used at five times the loading. Further, DBS-SCH_3_ showed a strong dependence of assembly on water content, in agreement with the *T*_gel_ measurements above (Figure 2A–C) and reflecting its greater hydrophobicity. For all LMWGs, there was a significant hysteresis between cooling and heating of ca. 60 °C, and in each case, the gels formed above a temperature of 0 °C, which would be required for the desired deicing/anti-icing applications.

**Figure 3:**
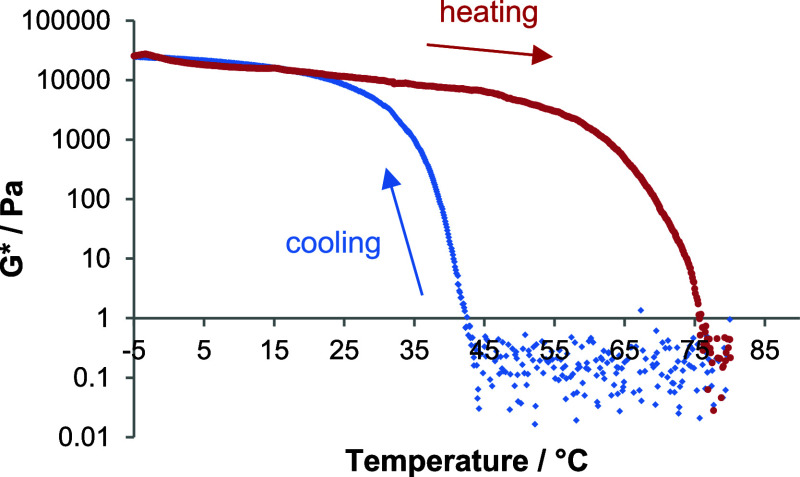
Variable temperature rheology of DBS in 75:25 MPG:H_2_O at a loading of 0.5 wt %/vol. Sample is cooled from 80 to −5 °C (blue) and then heated back to 80 °C (red) at a rate of 2 °C/min.

We then imaged each gel using scanning electron microscopy (SEM). We initially prepared samples in an analogous way to that which they would form in the application, by rapid cooling of a hot solution (98 °C) on an aluminum stub cooled to −21 °C. The sample was then placed in the freezer, and after 2 h, removed from the freezer and placed in a desiccator at room temperature to dry. As always, in SEM imaging, it is important to be aware that drying can potentially induce morphological change,^61^ but all samples were treated in the same way, and we reason this is a useful comparative imaging technique, that has been adjusted to be application-relevant.

For DBS, we observed uniform nanofibers, with diameters of ca. 5 nm, with some bundling of individual fibers (Figure 4, top left). Broadly, images obtained from gels formed in 75:25 MPG:H_2_O (Figure S19) and 50:50 MPG:H_2_O (Figure 4) were similar. Fibers formed by DBS-OCH_3_ were also similar in morphology (Figure 4, center left) and showed little difference dependent on precise solvent composition (Figure S20). In 50:50 MPG:H_2_O, DBS-SCH_3_ had a similar morphology with 5 nm nanofibers being observed (Figure 4, bottom left, Figure S22). On reducing the water content, the nanofibers of DBS-SCH_3_ became somewhat wider, with diameters of ca. 5–8 nm in 75:25 MPG:H_2_O and ca. 10–15 nm in 100% MPG (Figure S21).

**Figure 4:**
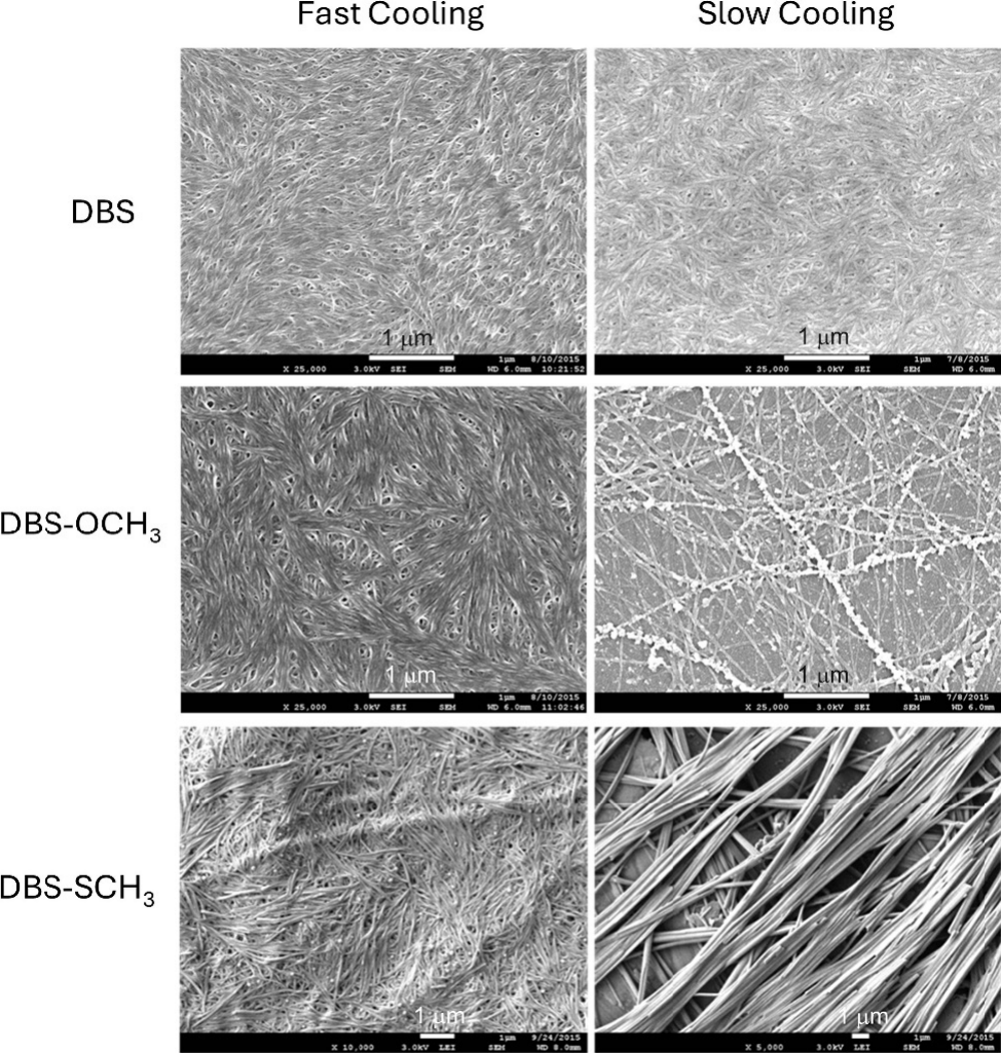
SEM images of DBS, DBS-OCH_3_, and DBS-SCH_3_ in 50:50 MPG:H_2_O showing the impact of gelator structure and cooling conditions on the observed morphology when gel formation is performed at (left) fast cooling freezing conditions and (right) ambient room temperature. All scale bars = 1 μm.

For comparison, we then prepared samples for imaging using a ‘slow cooling’ methodology, more typically used in academic LMWG analysis, in which gels were simply allowed to form slowly on the aluminum stub at room temperature. Interestingly, once a significant amount of water was present (50:50 MPG:H_2_O) there were some significant differences depending on the assembly pathway. On slow cooling of DBS-OCH_3_, it appeared that the nanofibers were beginning to show nodular signs of crystal formation (see Figure 4, center right). This may suggest that as the amount of water in the system increases, and the solubility is lower, there is a greater tendency of the gelator to separate from the solvent phase. Most interestingly, however, in 50:50 MPG:H_2_O the morphology of DBS-SCH_3_ changed dramatically, with a very different, more microcrystalline, morphology, composed of tape-like structures ca. 200–300 nm in diameter, that aggregate further into bundles ca. 1 μm in diameter (Figure 4, bottom right, Figure S22). We suggest that in the presence of a significant amount of water, the gelator is less compatible with the solvent—this is more marked for DBS-SCH_3_ than DBS-OCH_3_ because of its greater hydrophobicity. When DBS-SCH_3_ assembles rapidly, many fibers are quickly nucleated and small nanofibers with high surface area form. However, when DBS-SCH_3_ assembles slowly, larger tape-like crystalline structures are observed, formed from a smaller number of nucleation sites, and beneficially lowering the hydrophobic surface area. These different morphologies can be considered as kinetically favored (fast cooling) and thermodynamically favored (slow cooling) respectively.

The impact of water content on morphological switching in gels has been a topic of significant importance, and clearly DBS-SCH_3_ is susceptible to this kind of effect.^62^ Even more importantly though is the morphological difference induced by changing the gel preparation method—an important current field of research.^63–65^ This example constitutes a relatively rare example in which cooling rate directly impacts on the nanoscale outcome.^66–69^ Clearly, in the desired application, cooling rate will be rapid, as the hot LWMG solution is sprayed onto a very cold surface in cold ambient conditions—as such, the more desirable nanofibrillar morphology will be expected to predominate, but moving beyond previous theoretical work, these observations clearly demonstrate the importance gelation kinetics can potentially have when these materials are used in an applied setting.

### Gel Formation and Characterization in Deicing Fluid, DF+

Having probed the LMWGs in model MPG:H_2_O solvent mixtures, we then went on to formulate them into a commercial Type I deicing fluid (DF Plus [DF+], Kilfrost). Type I fluids contain 80% MPG, with the remaining 20% being composed of water and other additives, including a neutral, nonionic surfactant and dyes. Type I fluids contain no thickening products and are simply used to remove ice from surfaces. A Type I deicing fluid must provide a minimum ‘holdover time’ in the test rig (see below) of 3 min. In practical use, dilutions of this fluid with additional water are often applied, although caution must be applied depending on the severity of the ambient conditions.

We screened our preferred gelators as additives in DF+ at loadings of 1.0, 0.5, and 0.1 wt %/vol (Figure 5). To initially prepare the samples, a known weight of gelator was added to a 2 mL vial and 1 mL of DF+ was added. Dilutions were then also made in which DF+ was first combined with different amounts of water, as would typically be done in real-world use. For clarity, these dilutions are reported here based on the percentage of MPG that remains in the final sample, with undiluted DF+ therefore being “80%”. Each sample was sonicated for 30 min, then heated in an oil bath to 98 °C until a clear homogeneous solution was formed. Pleasingly, the LMWGs had solubility in the commercial DF+ product that was equivalent to the solubility in pure solvent mixtures.

**Figure 5:**
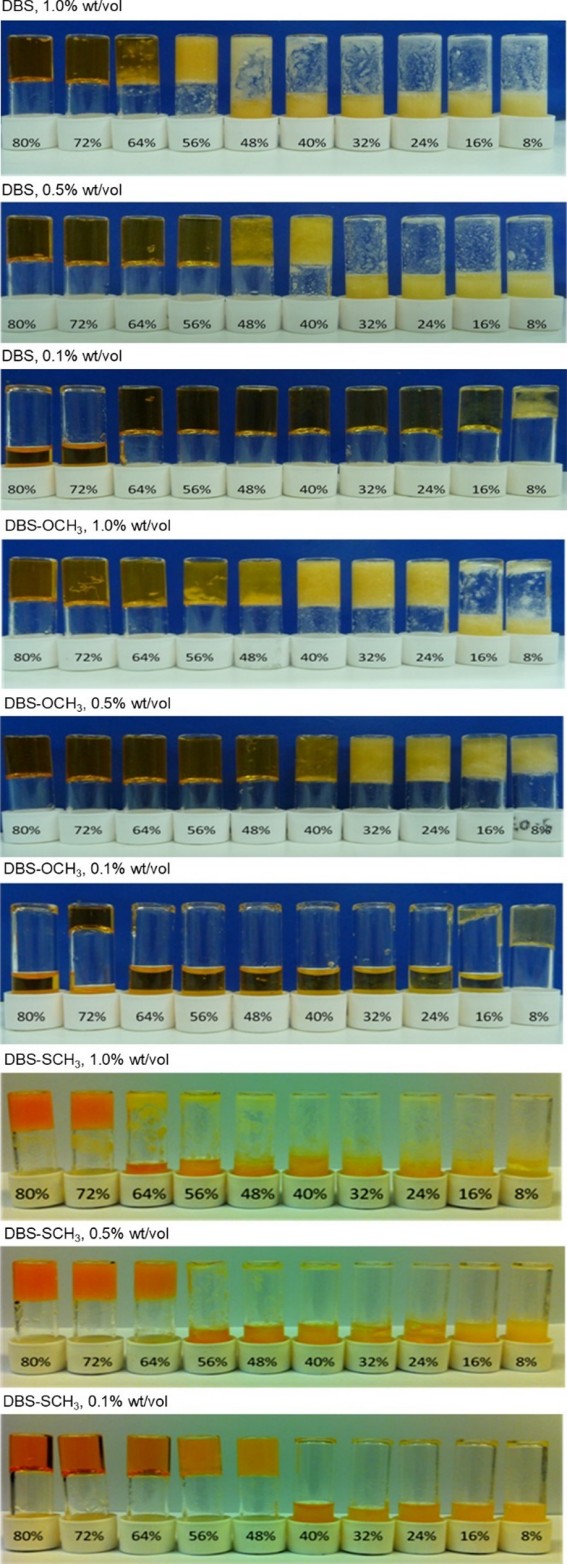
Initial screening of LMWGs in Type I DF+ and dilutions with different amounts of water for DBS, DBS-OCH_3_, and DBS-SCH_3_ at high (1.0 wt %/vol), medium (0.5 wt %/vol), and low (0.1 wt %/vol) concentrations.

At 1.0 wt %/vol, DBS formed fully transparent gels, down to 64:36 MPG:H_2_O, at 0.5 wt %/vol DBS formed gels to 48:52 MPG:H_2_O and at 0.1 wt %/vol DBS formed transparent gels to 16:84 MPG:H_2_O. The performance at 1.0 wt %/vol DBS can be compared to that described above in pure solvent, rather than DF+ (Table 1). DBS-OCH_3_ was generally more water-tolerant than DBS, reflecting the higher water solubility of this gelator. At 1.0 wt %/vol, DBS-OCH_3_ formed fully transparent gels, down to 48:52 MPG:H_2_O, and at 0.5 wt %/vol it formed gels to 40:60 MPG:H_2_O. However, at 0.1 wt %/vol, although DBS-OCH_3_ was soluble to 8:92 MPG:H_2_O, it struggled to establish a full sample-spanning gel-phase network, and only formed weak or partial gels. This is a result of the greater solubility of this gelator compared to DBS.

For DBS-SCH_3_, at high concentrations of 1.0 and 0.5 wt %/vol, the system did not fully dissolve, and although some gel-like materials were obtained with low amounts of H_2_O present, they were not transparent homogeneous gels. The SCH_3_ group clearly lowers LMWG solubility at higher loadings, especially in the presence of water. However, at 0.1 wt %/vol, DBS-SCH_3_ dissolved fully, and clear homogeneous gels were formed down to 48:52 MPG:H_2_O.

We then determined the minimum gelation concentrations (MGCs) of these gelators in DF+ at different dilutions (Table 3). In general, DBS and DBS-OCH_3_ had similar MGC values, although DBS was slightly more effective as the amount of water increased. In contrast, DBS-SCH_3_ formed gels at significantly lower MGCs (as low as 0.02 wt %/vol), but could only tolerate solvent mixtures down to 48:52 MPG:H_2_O before it became too insoluble to establish a gel network.

**Table 3:** Minimum Gelation Concentrations of Gelators in DF+ at Different Dilutions, Where the Percentage Refers to the Percentage of MPG in the Diluted Form of the Product, *N* = 3

	gelator/% wt/vol		
% MPG	DBS	DBS-OCH_3_	DBS-SCH_3_
80% (neat DF+)	0.2	0.2	0.1
72%	0.2	0.2	0.04
64%	0.1	0.2	0.04
56%	0.1	0.2	0.02
48%	0.1	0.2	0.02
40%	0.1	0.2	insoluble
32%	0.1	0.2	insoluble
24%	0.1	0.2	insoluble
16%	0.1	0.2	insoluble
8%	insoluble	0.2	insoluble

We then determined the thermal stabilities of the gels formed in DF+ at different LMWG concentrations (Figure S2). The thermal stability increased as the percentage of water increased, consistent with the results in neat MPG:H_2_O presented above (Figure 2A–C). As for what was observed in mixtures of pure solvent, this ‘H_2_O effect’ was most significant for DBS-SCH_3_, consistent with the view that the self-assembly of this gelator is particularly sensitive to water on grounds of its greater hydrophobicity, which is also what drives much more effective gel assembly at lower loadings.

We did consider performing a Hansen parameter analysis of these three different LMWGs based on this richer data. However, the assumptions about the HSPs of the solvent are less robust in DF+ that contains a variety of additives, and furthermore, it was hard to know how to account for the fact that the different LMWGs studied here worked at different concentrations. However, it is clear that as δ_p_ and δ_h_ increase significantly on the addition of more water into the DF+ product, solvophobically driven gelation improves, until such a critical point as the insolubility becomes too high and the formation of homogeneous gels becomes impossible. For each gelator this critical point can be considered as the ‘water sensitivity’ of the gelator at the stated concentration.

Gel formation was then scaled-up to 200 mL in glass Schott bottles (Figures S3–S5). The main change in terms of sample preparation was to heat samples overnight, rather than just for 1 h. In general, on scale-up, samples were occasionally less-able to support vial inversion, primarily because the much larger sample size means sample inversion creates a greater load at the interface of the gel, perhaps compounded by minor issues achieving complete LMWG solubilization at larger scale. It should be noted that this is not a limitation in terms of the intended application, as ultimately, samples would be sprayed onto aircraft as hot solutions (see below) and would form gels as thin films supported on the aircraft wing/body. Although these gels will be made at large scale, there is no requirement at any stage for bulk-scale gels that support their own weight on inversion.

We performed rheological analysis of the scaled-up samples produced in DF+, along with their aqueous dilutions (Table 4 and Figures S9–S14). Monitoring *G*′ indicated that in some cases, gel stiffness was significantly different in these DF+ systems. For DBS, the *G*′ increased from 3800 in 75:25 MPG:H_2_O to ca. 40,000 Pa for the equivalent solvent mix in DF+. On increasing the water content in DF+, the stiffness then decreased, in contrast to what was observed in neat MPG:H_2_O. By 50% MPG the *G*′ value for DBS was similar in both the pure solvent mix and the DF+ dilution. This clearly indicates that the other additives in the DF+ formulation have impacts on the rheological performance of DBS, enhancing the stiffness of the gel network. It is most likely that the neutral, nonionic surfactant is having this effect—there is considerable interest in studying the impacts of surfactants on LMWG assembly (and vice versa).^70–73^ This effect is more marked when less water is present—the *G*′ value falls significantly once water exceeds ca. 40%.

**Table 4:** Stiffness (*G*′) and Yield Stress (%) for Gels Formed by DBS (0.5 wt %/vol), DBS-OCH_3_ (0.5 wt %/vol), and DBS-SCH_3_ (0.1 wt %/vol) at Different Dilutions of DF+^a^

	DBS 0.5 wt %/vol		DBS-OCH_3_ 0.5 wt %/vol		DBS-SCH_3_ 0.1 wt %/vol	
% MPG	*G*′ (Pa)	yield stress (%)	*G*′ (Pa)	yield stress (%)	*G*′ (Pa)	yield stress (%)
80% (neat DF+)	35,400	0.6%	12,700	1.3%	13	1.3%
72%	43,800	0.8%	7700	0.7%	83	0.5%
64%	26,700	1.0%	7400	0.4%	500	3.2%
56%	3300	0.4%	3200	0.6%	450	3.2%
48%	4000	0.5%	900	0.5%	150	2.0%

a *N* = 2, errors ± 5%.

For DBS-OCH_3_, the *G*′ values were lower in DF+ than in neat solvent mixtures. Further, with a *G*′ value of (e.g.) 12,500 Pa in 80:20 MPG:H_2_O, the DBS-OCH_3_ gels were also softer than those formed by DBS, again different to the studies in neat MPG:H_2_O described above where DBS-OCH_3_ formed the stiffer gel, suggesting additives in the DF+ formulation have specific effects depending on the structure of the gelator. Once again, as for DBS, dilution of the DF+ system decreased *G*′, unlike in neat MPG:H_2_O. Such effects could be mediated either through solubility differences or direct interactions between the LMWG and the other additives present in DF+. Uncovering the precise nature of these effects is beyond the scope of this study given the proprietary nature of the DF+ formulation.

In DF+, DBS-SCH_3_ behaved differently to DBS or DBS-OCH_3_. As the amount of water increased, so did the stiffness of the gel, rising to a maximum stiffness at 56:44 MPG:H_2_O. This is similar to its behavior in neat solvent, where hydrophobic assembly yielded an optimum gel structure. The values of *G*′ remain relatively low for DBS-SCH_3_ consistent with the lower loading (0.1 wt %/vol) of this LMWG.

We then performed temperature-dependent rheology (Table S3 and Figures S15–S17). In each case, increasing the water content raised the thermal stability of the gel as expected. This suggests these gels are still hydrophobically driven in DF+. Although the additives present in the DF+ formulation appear to have some impact on gel stiffness, they therefore have less of an impact on thermal stability. The thermal behavior means these systems are optimal for application through spraying at temperatures of 65–100 °C and should form gels on a cold surface.

Time-resolved rheology was performed by applying hot samples to the rheometer plate set at 20 °C and the evolution of the gel over time was observed. As the amount of water in the system increased the time required for gel-assembly was lower (Table 5 and Figure S18), demonstrating hydrophobically driven self-assembly enhancing the gelation kinetics as well as their thermodynamics. DBS achieved more rapid gelation than DBS-OCH_3_, consistent with its greater hydrophobicity. DBS and DBS-OCH_3_ have practical gel forming kinetics at 20 °C, with gelation times ranging from 4.5 to 8 min for DBS and 4–15 min for DBS-OCH_3_. DBS-SCH_3_ formed gels more slowly unless a significant amount of water was present, but was being applied at a significantly lower loading. Obviously, in the proposed application, the surface to be deiced/anti-iced would be closer to 0 °C, so gelation kinetics would be enhanced. Indeed, in our holdover time studies (see below), where the gel solution was applied to a cold plate, DBS-SCH_3_ did not have problems with gel-forming kinetics.

**Table 5:** Time Taken in Minutes to Establish a Full Gel Network for DBS (0.5 wt %/vol), DBS-OCH_3_ (0.5 wt %/vol), and DBS-SCH_3_ (0.1 wt %/vol) in Different Dilutions of DF+, Where the Percentage Refers to the Percentage of MPG in the Diluted Form of the Product^a^

	gelator/% wt/vol		
% MPG	DBS (0.5%)	DBS-OCH_3_ (0.5%)	DBS-SCH_3_ (0.1%)
80% (neat DF+)	N/A	N/A	N/A
72%	7.9 min	14.3 min	N/A
64%	6.3 min	11.0 min	42 min
56%	4.9 min	7.3 min	8.8 min
48%	4.6 min	7.3 min	4.4 min
40%	insoluble	4.1 min	insoluble

a *N* = 2, errors ± 1.0 min.

Scanning electron microscopy performed on these gelators in DF+ at different dilutions indicated similar morphologies to those formed in neat solvent, suggesting that the other parts of the formulation of the commercial deicing product do not adversely impact on gelator self-assembly (Figures S23–S25). In the diluted DF+ formulation with 48% MPG, DBS-SCH_3_ once again formed different morphologies dependent on the rate of cooling, with much smaller nanofibers being formed on rapid cooling, typical of the application itself, and larger, microcrystalline objects being observed when samples were cooled slowly (Figure S25).

### Gelator Testing under Conditions Relevant for Aviation Deicing Applications

A deicing or anti-icing fluid must pass regulatory approval. One key specification is the water spray endurance test (WSET),^39,40^ a laboratory-based test to evaluate the holdover performance of fluids under different conditions. The test is performed in a temperature-controlled climatic chamber where the air temperature is held at −5 °C. Within the chamber, an aluminum frosticator plate, representative of the leading edge on an aircraft wing, is set to −5 °C and tilted at an angle of 10°. The plate consists of six test panels: four panels are used for the samples, while the outer two panels are used as controls. A motor travels across the frosticator, spraying a fine mist of water, which on freezing produces 5 ± 0.2 gdm^–2^ h of ice (the ‘catch’), replicating typical freezing conditions. Test samples are poured across the top of the panel ensuring the leading edge is fully covered—the fluid is thinnest at the top of the plate and thickest at the bottom, consequently, ice formation initiates at the top of the plate and progressively moves down. Once the first shard of ice reaches a distance of 2.5 cm from the top of the plate, the time is recorded, and the holdover time is calculated (Table S4) relative to the catch. Only samples that formed gels or partial gels in the testing above were investigated for holdover time.

Using this industry-standard approach, we determined the performance of our gels. Samples were applied to the panel when hot (95 °C) as they could not be poured at room temperature. In consumer use, deicing products are diluted prior to application, with the degree of dilution depending on the outside air temperature. For DF+ in the absence of an LMWG additive (industry standard control), a holdover time of ca. 7 min was observed in this test, exceeding the minimum requirement of 3 min for a Type I fluid (Table S5 and Figure 6). As DF+ was diluted, this holdover time decreases, as would be expected, because the glycol content of the relatively simple fluid decreases and it is therefore less effective at preventing ice build-up.

**Figure 6:**
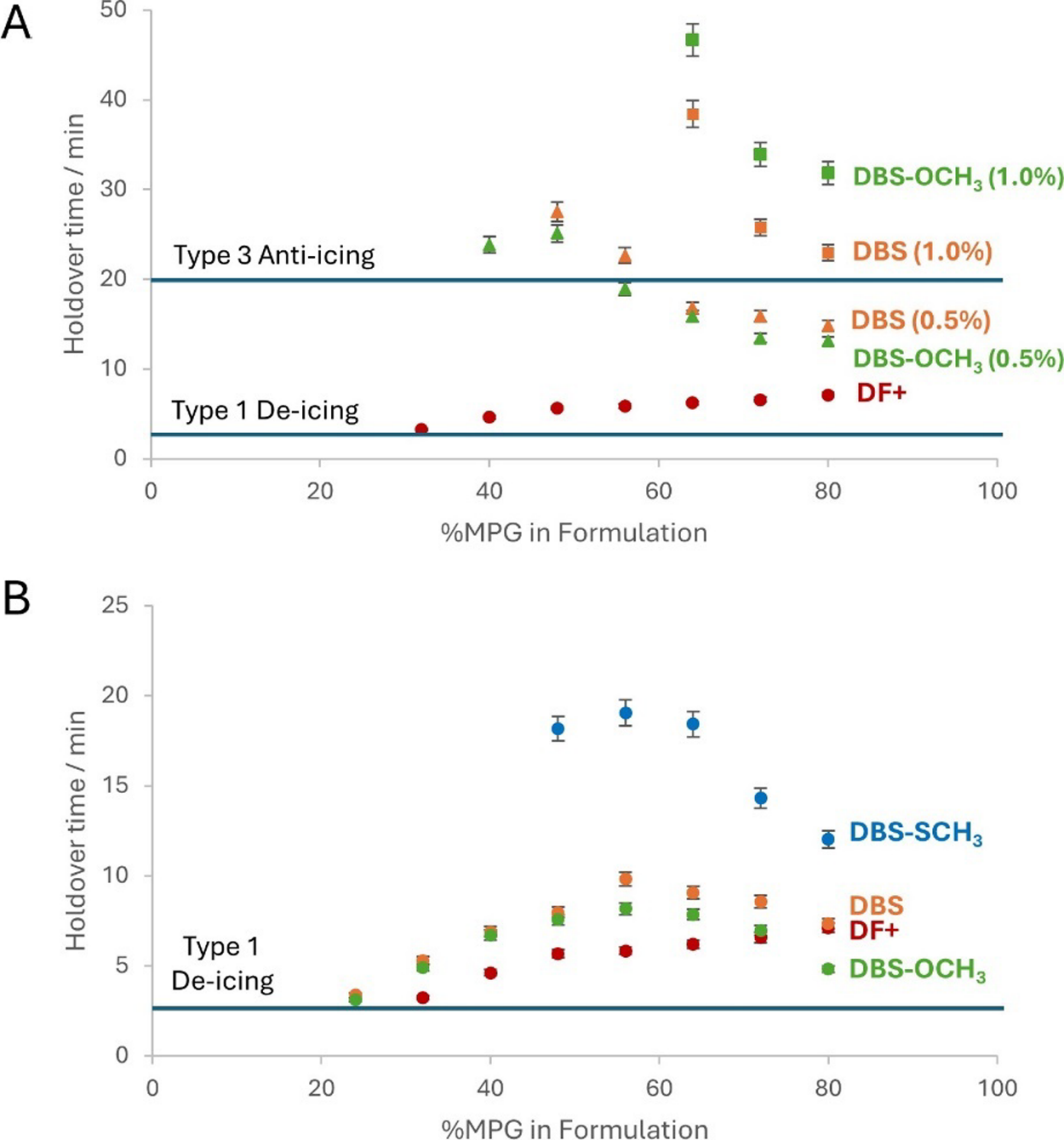
Summary of holdover time performance of DF+ and LMWGs formulated into DF+ at different dilutions. (A) At loadings of 1.0 and 0.5% comparing DBS (orange) and DBS-OCH_3_ (green) formulated into DF+ at different dilutions with water and compared with unmodified deicing fluid DF+ (red). (B) At loadings at a loading of 0.1 wt %/vol DBS (orange), DBS-OCH_3_ (green), and DBS-SCH_3_ (blue) formulated into DF+ at different dilutions with water and compared with the baseline performance of the deicing fluid DF+ (red).

The holdover times for the gelators at different dilutions of DF+ were determined (Table 6 and Figure 6), with DBS and DBS-OCH_3_ being tested at loadings of 1.0, 0.5, and 0.1 wt %/vol, while DBS-SCH_3_, which is less soluble at higher loadings, was only tested at 0.1 wt %/vol. Considering DBS as an LMWG additive, it is evident that it improves the performance of neat DF+ at all loadings. Impressively, at 1.0 wt %/vol, DBS increases the holdover time of DF+ ca. 3 fold, with the resulting fluid performing like a Type III anti-icing fluid with a holdover time >20 min (Figure 6A). At lower loadings of DBS, the holdover time increases less, but the gels are better able to withstand dilution and still assemble effectively, therefore with 0.5 wt %/vol DBS, Type III anti-icing holdover performance is achieved with a lower glycol content.

**Table 6:** Holdover Time Performance (WSET Test, min) of Neat Type 1 Deicing Fluid (DF+) Combined with LMWGs at Different Loadings and Varied Dilutions with Water, *N* = 2

	holdover times for different combinations of LMWG and diluted DF+ (min)						
	DBS			DBS-OCH_3_			DBS-SCH_3_
% MPG	1 wt %/vol	0.5 wt %/vol	0.1 wt %/vol	1 wt %/vol	0.5 wt %/vol	0.1 wt %/vol	0.1 wt %/vol
80%	22.95 ± 0.87	14.82 ± 0.58	7.35 ± 0.28	31.83 ± 1.23	13.10 ± 0.50	4.83 ± 0.18	12.03 ± 0.47
72%	25.77 ± 0.90	15.88 ± 0.62	8.58 ± 0.33	33.90 ± 1.32	13.47 ± 0.52	6.98 ± 0.27	14.33 ± 0.55
64%	38.40 ± 1.47	16.77 ± 0.65	9.08 ± 0.33	46.68 ± 1.78	15.88 ± 0.62	7.87 ± 0.30	18.43 ± 0.72
56%	X	22.63 ± 0.88	9.82 ± 0.38	X	18.93 ± 0.73	8.18 ± 0.32	19.05 ± 0.73
48%	X	27.53 ± 1.07	7.98 ± 0.28	X	25.12 ± 0.97	7.57 ± 0.30	18.18 ± 0.70
40%	X	X	6.92 ± 0.27	X	23.82 ± 0.93	6.73 ± 0.27	X
32%	X	X	5.30 ± 0.22	X	X	4.92 ± 0.18	X
24%	X	X	3.38 ± 0.13	X	X	3.13 ± 0.12	X
16%	X	X	X	X	X	X	X

Pleasingly, as the glycol content is decreased by dilution, and the water content increases, the holdover time in the presence of DBS increases—this is in contrast to the normal decrease in performance as glycol content decreases. Indeed, in general, in the presence of all LMWGs (at all loadings), the holdover time reaches a maximum at ca. 50:50 MPG:H_2_O and then decreases again as the amount of water increases further. This reflects the fact that as more water is introduced to DF+, the LMWG is better able to establish a solid-like network by hydrophobically driven assembly. However, once the amount of water becomes too large, the solubility of the LMWG becomes too low for it to establish such an effective sample spanning network. This latter point is the reason why lower loadings of DBS are better able to form gels and hence improve holdover times at higher dilutions. This ability of LMWG-loaded samples to achieve enhanced performance at higher dilutions could enable development of deicing/anti-icing products with lower glycol content, offering significant environmental benefits.

DBS-OCH_3_ improves the performance of DF+ significantly and, like DBS, at 0.5 wt %/vol can improve the performance of neat DF+ ca. 2–4 fold as well as enhancing performance at higher dilutions (Figure 6A and Table 6). The greater hydrophilicity of DBS-OCH_3_ compared to DBS gives it the capacity to tolerate even more dilution while still achieving good anti-icing performance.

At the very low loading of 0.1 wt %/vol, DBS-SCH_3_ has by far the best performance in terms of holdover time—impressively doubling (or more) the holdover time of DF+ (Figure 6B and Table 6), consistent with its highly effective hydrophobic gel assembly under these conditions as discussed above. However, as a disadvantage, the lower water solubility of DBS-SCH_3_ means it is less able to tolerate dilution than DBS or DBS-OCH_3_ and the glycol content has to remain ≥48%, whereas the other LMWGs operate to some extent down to glycol levels of 24%.

In summary, at 1.0 wt %/vol, DBS and DBS-OCH_3_ formulated in Type I deicing agent DF+ have the potential to extend the performance of the fluid, which acts instead as a Type III anti-icing agent. However, there are some limits on the dilutions at which the system can be applied. At 0.5 wt %/vol, DBS and DBS-OCH_3_ in DF+ once again have the potential to act as Type III anti-icing agents, if diluted to an appropriate level—this lower glycol content is also desirable on environmental grounds and may enable the formation of different types of anti-icing product. Even at 0.1 wt %/vol (Figure 6B), DBS-SCH_3_ has the potential to extend the performance of DF+ close to that of a Type III fluid, which given the low loading, and the low-cost of DBS systems, means it has significant advantages. Given that the remainder of the DF+ formulation has not, in this study, been optimized in any way for the presence of the LMWG, it is possible that highly effective anti-icing agents may be formulated using this approach.

The second important specification of anti-icing products for use in the aviation industry is that they should meet the aerodynamic acceptability test based on the ability of the anti-icing fluid to flow from the surface of an aircraft during acceleration and takeoff to leave an acceptable minimum amount of material on the wing. Unfortunately, the standard industry test kit could not be used, as the gelled fluids had to be applied hot, damaging the perspex box onto which they were loaded. We therefore applied a rheological test of the ability of these materials to breakdown under shear based on the AS9000 standard,^74^ which states that for an anti-icing fluid to be used effectively, it must demonstrate that it can be broken down by >74% of its original viscosity value.

We performed oscillatory rheometry and monitored the gel over time. Initially, the strain was set such that the structure is not damaged, allowing us to determine gel stiffness. At a certain time-point, a significant strain was applied (10, 50, or 100%). The shear rate applied at 100% strain is most similar to that experienced on aircraft takeoff. At a further time-point, the strain was then lowered again, allowing us to understand if the gels would rebuild and self-heal—an interesting potential feature of supramolecular gels.

We tested 0.5 wt %/vol DBS (Figure 7, top) and DBS-OCH_3_ in neat DF+ with 80% MPG. Both LMWGs showed similar behavior forming stiff structures that rapidly break down on application of shear. Pleasingly, on applying 10% strain, the *G*′ value falls to ca. 100 Pa, and with 50 or 100% strain, it rapidly falls even further to ca. 10 Pa and then progressively to ca. 1 Pa (Table S6). As such, the gelled DF+ meets the requirement for aerodynamic acceptability.

**Figure 7:**
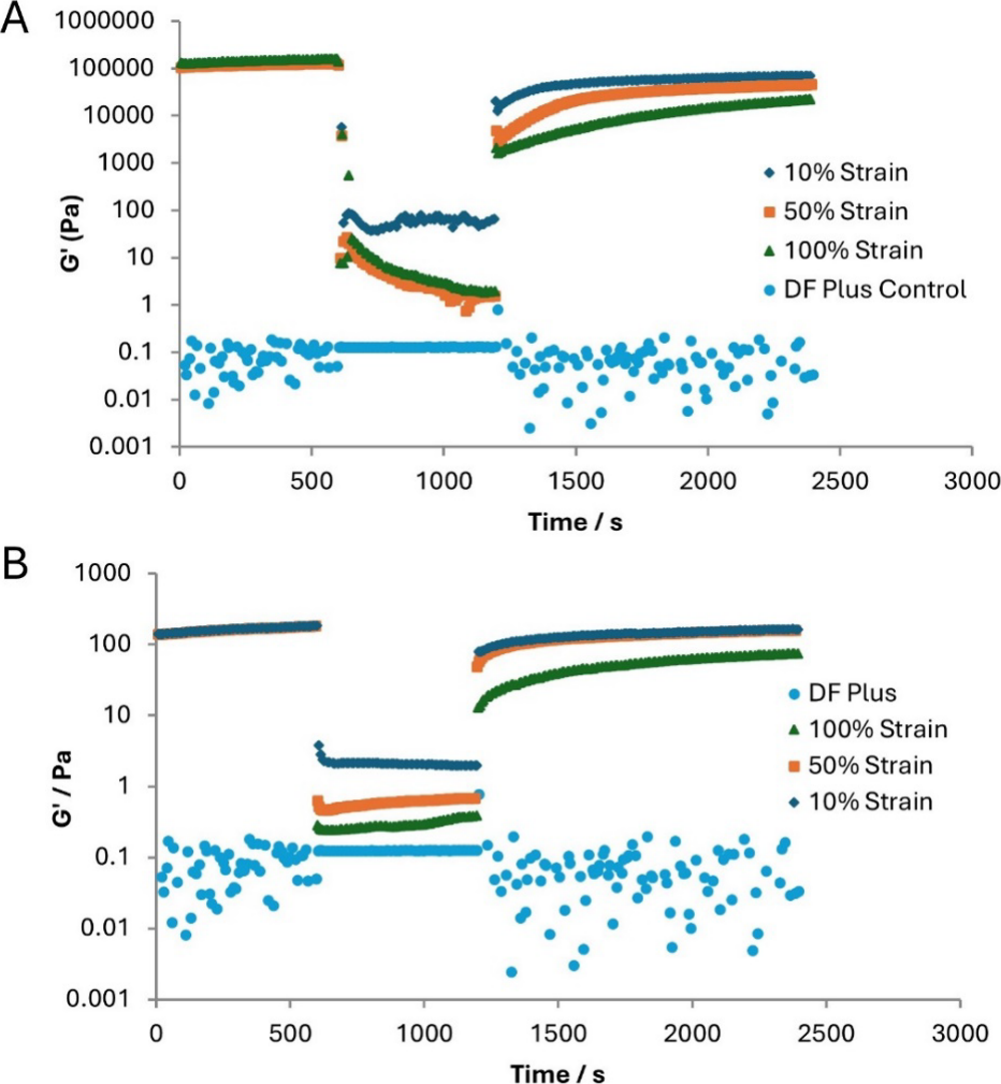
Rheology experiments on (A) DBS (0.5 wt %/vol) and (B) DBS-SCH_3_ (0.1 wt %/vol) in DF+ (80% MPG), in which strain is initially within the LVR, and then increases to 10, 50, or 100% after 10 min. Strain is applied for 10 min and the extent to which the gel is broken down is monitored by the decrease in *G*′. The strain is then reduced to a value within the LVR, and the ability of the gel to reform (self-heal) over time is measured.

On removal of strain, the gels partially recovered their rheological performance (Figure 7 and Table S7). When a 10% strain has been applied and then removed, the gel recovers 40–60% of its original *G*′ value. When a 100% strain is applied and removed, the gels can only rapidly rebuild ca. 10% of their original stiffness, and then slowly increase their stiffness further. It should be noted that in the experiment, there is no airflow to remove the solution from the plate when strain is applied—we anticipate that in the desired application, airflow would remove most of the sol from the aircraft wing, and hence the thixotropic nature of these gels should not cause a significant problem with gel rebuilding after takeoff.

We then tested 0.1 wt %/vol DBS-SCH_3_ in 80% DF+ (Figure 7, bottom). This gelator showed similar behavior to those described above, although the initial gel was much less stiff (ca. 100 Pa) as a result of the lower loading. Once again, application of strain broke down the structure of the gel, with increasing strain doing this more effectively (Table S6). On removal of strain, the DBS-SCH_3_ gels were better able to self-heal (Table S7), regaining up to 80% of their original stiffness (higher strain leads to lower gel recovery), reflecting the excellent ability of DBS-SCH_3_ to self-assemble. The lower starting stiffness of this LMWG means it should be even more easily removed on the application of strain in combination with airflow in the desired application.

## Conclusions

In summary, DBS-based gels are effective thickeners for MPG/water mixtures, with potential to extend the application of such fluids from deicing to anti-icing. A range of derivatives was synthesized and tested, with optimal performance being observed for DBS, DBS-OCH_3_ and DBS-SCH_3_. Notably, DBS-SCH_3_, reported for the first time here, formed gels in these solvent mixtures at concentrations nearly 10-fold lower than either DBS or DBS-OCH_3_, making it a potent gelator.

The performance of the gels depends on the percentage of water present in the MPG—as the amount of water increases, gelation ability and thermal stability improve as the gelator solubility decreases and a ‘solid-like’ network can be more easily formed. However, once the water content reaches a certain level, gelator solubility becomes too low and gelation is prevented—this effect is more marked at higher gelator loadings, where solubility is more easily limited. SEM indicated that the LMWGs formed nanoscale fibrillar networks composed of fibers ca. 5 nm in diameter on rapid cooling. On slow cooling under thermodynamic control in 50:50 MPG:H_2_O, DBS-SCH_3_ formed an interesting alternative microcrystalline tape-like morphology.

The ability of DBS-SCH_3_ to self-assemble at such low concentration (<0.1 wt %/vol) suggests that this may be a valuable additive. Given the low-cost of DBS derivatives, and the current large-scale industrial applications of DBS itself, we suggest that DBS-SCH_3_ has considerable promise in both economic and environmental terms, by potentially lowering the amount of additive required in a variety of pre-existing applications.

With regard to anti-icing, the LMWGs were tested in a commercially available deicing fluid (DF+). Each gelator has some capability to extend the performance of the deicing fluid in the water spray endurance test (WSET), such that it has holdover times equivalent to those expected for a higher performance Type III anti-icing fluid. The application of strain typical of that experienced on aircraft takeoff led to the breakdown of the gels, suggesting aerodynamic acceptability. The precise details of performance depend on the choice of LMWG, its concentration, and the dilution at which the DF+ fluid is employed. In general, performance improves at higher dilutions up to 50:50 MPG:H_2_O, as the hydrophobically driven assembly of the gel is more effective in the presence of more water. This is in contrast to traditional deicing/anti-icing fluids, where performance improves with greater glycol content (i.e., less water). This suggests that LMWGs, as well as replacing polymer additives, may enable the formulation of anti-icing products with lower glycol content and greater environmental sustainability. Considering the three different LMWGs studied, if the end-user prioritizes an additive that operates at the lowest possible loadings, but with some restrictions on application conditions, DBS-SCH_3_ would be optimal. However, if the priority is to minimize glycol usage, then DBS-OCH_3_ is the most effective LMWG reported here. On the other hand, if the end-user prioritizes a low-cost additive that is already well-established in a range of applications and readily available on bulk scale, unmodified DBS would be optimal.

## Supplementary Material



## Notes

Materials and methods and further characterization data (PDF)

## References

[1] Weiss R. G. (2014). The Past,
Present, and Future of Molecular Gels. What is the Status of the Field
and Where is it Going?. J. Am. Chem. Soc..

[2] Draper E. R., Adams D. J. (2017). Low-Molecular-Weight
Gels: The State of the Art. Chem..

[3] Draper E. R., Adams D. J. (2024). Controlling Supramolecular
Gels. Nat. Mater..

[4] Thakur, V. K. ; Thakur, M. K. ; Voicu, S. I. , Eds.; Polymer Gels: Perspectives and Applications; Springer, 2018.

[5] Smith D. K. (2023). Supramolecular
Gels–A Panorama of Low-Molecular-Weight Gelators from Ancient
Origins to Next-Generation Technologies. Soft
Matter.

[6] Dresel, W. ; Heckler, R. P. Lubricating Greases. In Lubricants and Lubrication; Mang, T. ; Dresel, W. , Eds.; Wiley-VCH: Weinhem, 2017.

[7] Roehl, E. L. ; Tan, H. B. Solid Antiperspirant Composition and Process for its Preparation. US Patent 4,154,816, 1979.

[8] Ando, T. ; Yamazaki, Y. Adhesive Crayon Composition Containing Sorbitol-Benzaldehyde Reaction Product as Additive. US Patent 3,846,363, 1974.

[9] Hamada, K. ; Uchiyama, H. Polyolefin plastic compositions. US Patent 4,016,118, 1977.

[10] Uchiyama, H. Polyolefin resin composition comprising a dibenzylidene sorbitol derivative. US Patent 4,483,952, 1984.

[11] Rekers, J. W. Bis­(3,4-dialkylbenzylidene) Sorbitol Acetals and Compositions Containing Same. US Patent 5,049,605, 1991.

[12] Horváth Z., Gyarmati B., Manyhárd A., Doshev P., Gahleitner M., Varga J., Puzánsky B. (2014). The Role of Solubility and Critical
Temperatures for the Efficiency of Sorbitol Clarifiers in Polypropylene. RSC Adv..

[13] Wilder E. A., Wilson K. S., Quinn J. B., Skrtic D., Antonucci J. M. (2005). Effect
of an Organogelator on the Properties of Dental Composites. Chem. Mater..

[14] Karim, N. ; Jones, T. D. ; Lewandowski, K. M. ; Craig, B. D. ; Mitra, S. B. ; Yang, J. Dental Compositions Including Organogelators, Products, and Methods. US Patent 8,445,558, 2013.

[15] Breton, M. P. ; Boils-Boissier, D. C. ; Titterington, D. R. ; Thomas, Jr., J. W. ; Banning, J. H. ; Bedford, C. E. ; Wuest, J. D. Phase Change Inks Containing Gelator Additives. US Patent 6,872,243, 2005.

[16] Yamazaki M., Jemcov A., Sakaue H. (2021). A Review on
the Current Status of
Icing Physics and Mitigation in Aviation. Aerospace.

[17] Rekuviene R., Saeidiharzand S., Mazeika L., Samaitis V., Jankauskas A., Sadaghiani A. K., Gharib G., Muganli Z., Kosar A. (2024). A Review on
Passive and Active Anti-icing and De-icing Technologies. Appl. Thermal Eng..

[18] Wu, Z. ; Wang, Q. Effect of and Protection from Ice Accretion on Aircraft. In Ice Adhesion: Mechanism, Measurement and Mitigation; Mittal, K. L. ; Choi, C.-H. , Eds.; Scrivener Publishing LLC, 2020; pp 577–606.

[19] Grishaev V. G., Borodulin I. S., Usachev I. A., Amirfazli A., Drachev V. P., Rudenko N. I., Gattarov R. K., Bakulin I. K., Makarov M. V., Akhatov I. S. (2021). Anti-icing Fluids Interaction with
Surfaces: Ice Protection and Wettability Change. Int. Commun. Heat Mass Transfer.

[20] Zhuo Y., Chen J., Xiao S., Li T., Wang F., He J., Zhang Z. (2021). Gels as Emerging Anti-icing
Materials: A Mini Review. Mater. Horiz..

[21] Urata C., Dunderdale G. J., England M. W., Hozumi A. (2015). Self-lubricating Organogels
(SLUG) with Exceptional Syneresis-induced Anti-sticking Properties
Against Viscous Emulsions and Ices. J. Mater.
Chem. A.

[22] Urata C., Hönes R., Sato T., Kakiuchida H., Matsuo Y., Hozumi A. (2019). Textured Organogel
Films Showing
Unusual Thermoresponsive Dewetting, Icephobic, and Optical Properties. Adv. Mater. Interfaces.

[23] Yu Y., Jin B., Jamil M. I., Cheng D., Zhang Q., Zhan X., Chen F. (2019). Highly Stable
Amphiphilic Organogel with Exceptional Anti-Icing Performance. ACS Appl. Mater. Interfaces.

[24] Ru Y., Fang R., Gu Z., Jiang L., Liu M. (2020). Reversibly
Thermosecreting Organogels with Switchable Lubrication and Anti-Icing
Performance. Angew. Chem., Int. Ed..

[25] Jin Y., Wu C., Yang Y., Wu J., He Z., Wang J. (2020). Inhibiting
Condensation Freezing on Patterned Polyelectrolyte Coatings. ACS Nano.

[26] Wang Z., Lin B., Sheng S., Tan S., Wang P., Tao Y., Liu Z., He Z., Wang J. (2022). Bioinspired Anti-Icing Hydrogel Enabled
by Ice-Nucleating Protein. CCS Chem..

[27] Zhang Y., Yan W., Lin Y., Zhu J., Zhao H., Li T. (2024). Multifunctional
Anti-Icing Gel Surface with Enhanced Durability. ACS Appl. Mater. Interfaces.

[28] Zeng J., Yin Y., Zhang L., Hu W., Zhang C., Chen W. (2016). A Supraomlecular
Gel Approach to Minimize the Neural Cell Damage during Cryopreservation
Process. Macromol. Biosci..

[29] Lan D., Chen X., Li P., Zou W., Wu L., Chen W. (2018). Using a Novel Supramolecular Gel
Cryopreservation System in Microchannel
to Minimize the Cell Injury. Langmuir.

[30] Murray K. A., Gibson M. I. (2022). Chemical Approaches
to Cryopreservation. Nat. Rev. Chem..

[31] Jones C. D., Steed J. W. (2016). Gels with Sense:
Supramolecular Materials that Respond
to Heat. Light and Sound. Chem. Soc. Rev..

[32] Raeburn J., Cardoso A. M., Adams D. J. (2013). The Importance
of the Self-assembly
Process to Control Mechanical Properties of Low Molecular Weight Hydrogels. Chem. Soc. Rev..

[33] Lan Y., Corradini M. G., Weiss R. G., Raghavan S. R., Rogers M. A. (2015). To Gel
or Not To Gel: Correlating Molecular Gelation with Solvent Parameters. Chem. Soc. Rev..

[34] Yamasaki S., Tsutsumi H. (1996). The Thermal Behavior of 1,3:2,4-Di-*O*-benzylidene-D-sorbitol/Ethylene Glycol Gel. Bull. Chem. Soc. Jpn..

[35] Kumar D. K., Jose D. A., Das A., Dastidar P. (2005). First Snapshot of a
Nonpolymeric Hydrogelator Interacting with its Gelling Solvents. Chem. Commun..

[36] Wang Y., Wu Y., Yu Q., Zhang J., Ma Z., Zhang M., Zhang L., Bai Y., Cai M., Zhou F., Liu W. (2020). Significantly Reducing
Friction and Wear of Water-Based Fluids with
Shear Thinning Bicomponent Supramolecular Hydrogels. Adv. Mater. Interfaces.

[37] Okesola B. O., Vieira V. M. P., Cornwell D. J., Whitelaw N. K., Smith D. K. (2015). 1,3:2,4-Dibenzylidene-D-Sorbitol
(DBS) and its Derivatives–Efficient, Versatile and Industrially-relevant
Low-molecular-weight Gelators with over 100 Years of History and a
Bright Future. Soft Matter.

[38] Okesola B. O., Smith D. K. (2013). Versatile Supramolecular
pH-tolerant Hydrogels which
Demonstrate pH-dependent Selective Adsorption of Dyes from Aqueous
Solution. Chem. Commun..

[39] G-12ADF Aircraft Deicing Fluids, Water Spray and High Humidity Endurance Test Methods for AMS1424 and AMS1428 Aircraft Deicing/Anti-icing Fluids; SAE International, 2019.

[40] Laforte J.-L., Louchez P., Bouchard G., Ma F. (1990). A Facility to Evaluate
Performance of Aircraft Ground De/Anit-icing Fluids Subjected to Freezing
Rain. Cold Regions Sci. Technol..

[41] Li J., Fan K., Guan X., Yu Y., Song J. (2014). Self-Assembly Mechanism
of 1,3:2,4-Di­(3,4-Dichlrobenzylidene)-d-Sorbitol and Control
of the Supramolecular Chirality. Langmuir.

[42] Yamasaki S., Tsutsumi H. (1995). The Dependence of the
Polarity of Solvents on 1, 3:2,
4-Di-O-benzylidene-D-sorbitol Gel. Bull. Chem.
Soc. Jpn..

[43] Watase M., Nakatani Y., Itagaki H. (1999). On the Origin of the Formation and
Stability of Physical Gels of Di-*O*-benzylidene-d-sorbitol. J. Phys. Chem. B.

[44] Wilder E. A., Spontak R. J., Hall C. K. (2003). The molecular structure and intermolecular
interactions of 1,3:2,4-dibenzylidene-D-sorbitol. Mol. Phys..

[45] Diehn K. K., Oh H., Hashemipour R., Weiss R. G., Raghavan S. R. (2014). Insights into Organogelation
and its Kinetics from Hansen Solubility Parameters. Toward *a priori* Predictions of Molecular Gelation. Soft Matter.

[46] Lan Y., Corradini M. G., Liu X., May T. E., Borondics F., Weiss R. G., Rogers M. A. (2014). Comparing and correlating solubility
parameters governing the self-assembly of molecular gels using 1,3:2,4-dibenzylidene
sorbitol as the gelator. Langmuir.

[47] Singh A., Auzanneau F.-I., Corradini M. G., Grover G., Weiss R. G., Rogers M. A. (2017). Molecular
Nuances Governing the Self-Assembly of 1,3:2,4-Dibenzylidene-d-sorbitol. Langmuir.

[48] Nasr P., Corradini M. G., Hill J., Read S. T., Rosendahl S. M., Weiss R. G., Auzanneau F.-I., Rogers M. A. (2020). Hansen Solubility
Parameters Clarify the Role of the Primary and Secondary Hydroxyl
Groups on the Remarkable Self-Assembly of 1:3,2:4-Dibenzylidene Sorbitol. J. Phys. Chem. C.

[49] Feng R., Chen L., Hou Z., Song J. (2007). Synthesis
of Dibenzylidene
Sorbitol Series Compound. Trans. Tianjin Univ..

[50] Stan R., Ott C., Sulca N., Lungu A., Iovu H. (2009). Functionalized D-Sorbitol-Based
Organogelators for Dental Materials (I). Mater.
Plast..

[51] Stan R., Rosca S., Ott C., Rosca S., Perez E., Rico-Lattes I., Lattes A. (2006). D-Sorbitol-Based Organogelators with
Nitrogen Groups. Rev. Roum. Chim..

[52] Cornwell D. J., Daubney O. J., Smith D. K. (2015). Photopatterned
Multidomain Gels:
Multi-Component Self-Assembled Hydrogels Based on Partially Self-Sorting
1,3:2,4-Dibenzylidene-d-sorbitol Derivatives. J. Am. Chem. Soc..

[53] Abbott, S. ; Hansen, C. M. Hansen Solubility Parameters in Practice; CRC Press, 2008.

[54] Rosa
Nunes D., Raynal M., Isare B., Albouy P.-A., Bouteiller L. (2018). Organogel Formation Rationalized by Hansen Solubility
Parameters: Improved Methodology. Soft Matter.

[55] Yan N., Xu Z., Diehn K. K., Raghavan S. R., Fang Y., Weiss R. G. (2013). How Do
Liquid Mixtures Solubilize Insoluble Gelators? Self-Assembly Properties
of Pyrenyl-Linker-Glucono Gelators in Tetrahydrofuran-Water Mixtures. Langmuir.

[56] Bonnet J., Suissa G., Raynal M., Bouteiller L. (2015). Organogel
Formation Rationalized by Hansen Solubility Parameters: Influence
of Gelator Structure. Soft Matter.

[57] Antonijević I.
S., Janjić G. V., Milčić M. K., Zarić S. D. (2016). Preferred
Geometries and Energies of Sulfur–Sulfur Interactions in Crystal
Structures. Cryst. Growth Des..

[58] Vogel L., Wonner P., Huber S. M. (2019). Chalcogen
Bonding: An Overview. Angew. Chem., Int. Ed..

[59] Liao L., Jia X., Lou H., Zhong J., Lu H., Ding S., Chen C., Hong S., Luo X. (2021). Supramolecular Gel
Formation Regulated by Water Content in Organic Solvents: Self-assembly
Mechanism and Biomedical Applications. RSC Adv..

[60] Dawn A., Kumari H. (2018). Low Molecular Weight Supramolecular Gels Under Shear:
Rheology as the Tool for Elucidating Structure-Function Correlation. Chem.—Eur. J..

[61] Adams D. J. (2018). Does Drying
Affect Gel Networks?. Gels.

[62] Liu C., Jin Q., Lv K., Zhang L., Liu M. (2014). Water Tuned the Helical
Nanostructures and Supramolecular Chirality in Organogels. Chem. Commun..

[63] Tantakitti F., Boekhoven J., Wang X., Kazantsev R., Yu T., Li J., Zhuang E., Zandi R., Ortony J. H., Newcomb C. J., Palmer L. C., Shekhawat G. S., Olvera de la Cruz M., Schatz G. C., Stupp S. (2016). I. Energy landscapes
and functions of supramolecular systems. Nat.
Mater..

[64] Debnath S., Roy S., Abul-Haija Y. M., Frederix P. W. J. M., Ramalhete S. M., Hirst A. R., Javid N., Hunt N. T., Kelly S. M., Angulo J., Khimyak Y. Z., Ulijn R. V. (2019). Tunable
Supramolecular Gel Properties by Varying Thermal History. Chem.—Eur. J..

[65] Yuan S. C., Lewis J. A., Sai H., Weigand S. J., Palmer L. C., Stupp S. I. (2022). Peptide Sequence Determines Structural Sensitivity
to Supramolecular Polymerization Pathways and Bioactivity. J. Am. Chem. Soc..

[66] Coates I. A., Smith D. K. (2009). Controlled Self-Assembly—Synthetic Tunability
and Covalent Capture of Nanoscale Gel Morphologies. Chem.—Eur. J..

[67] Cui J., Liu A., Guan Y., Zheng J., Shen Z., Wan X. (2010). Tuning the
Helicity of Self-Assembled Structure of a Sugar-Based Organogelator
by the Proper Choice of Cooling Rate. Langmuir.

[68] Nebot V. J., Diaz-Oltra S., Smets J., Fernandez Prieto S., Miravet J. F., Escuder B. (2014). Freezing Capture
of Polymorphic Aggregates
of Bolaamphiphilic L-Valine-Based Molecular Hydrogelators. Chem.—Eur. J..

[69] Du S., Jiang Y., Jiang H., Zhang L., Liu M. (2024). Pathway-Dependent
Self-Assembly for Control over Helical Nanostructures and Topochemical
Photopolymerization. Angew. Chem., Int. Ed..

[70] Brizard A., Stuart M., van Bommel K., Friggeri A., de Jong M., van Esch J. (2008). Preparation of Nanostructures
by Orthogonal Self-Assembly
of Hydrogelators and Surfactants. Angew. Chem.,
Int. Ed..

[71] Xu Y., Laupheimer M., Preisig N., Sottmann T., Schmidt C., Stubenrauch C. (2015). Gelled Lyotropic
Liquid Crystals. Langmuir.

[72] Steck K., Van Esch J. H., Smith D. K., Stubenbrauch C. (2019). Tuning gelled
lyotropic liquid crystals (LLCs)–probing the influence of different
low molecular weight gelators on the phase diagram of the system H_2_O/NaCl–Genapol LA070. Soft Matter.

[73] Su L., Mabesoone M. F. J., Schoenmakers S. M. C., Muller C., Vleugels M. E. J., Dhiman S., Wijker S., Palmans A. R. A., Meijer E. W. (2022). Dilution-induced
Gel-Sol-Gel-Sol Transitions by Competitive Supramolecular Pathways
in Water. Science.

[74] AS5900E Standard Test Method for Aerodynamic Acceptance of AMS1424 and AMS1428 Aircraft Deicing/Anti-Icing Fluids; SAE International, 2021.

