# Supplementary material for: Supramolecular Gels with Potential Applications as Anti-Icing Agents
Source: Langmuir. 2025 May 23;41(22):13932–47. doi: 10.1021/acs.langmuir.5c00755 (PMC12164336; doi:10.1021/acs.langmuir.5c00755)
Supplement: Supplementary file 1 [file la5c00755_si_001.pdf]

Supporting Information

Supramolecular Gels with Potential Applications as Anti-icing Agents

Nicole K. McLeod,[a,b] *Lee Stokes,*[b] *Jerry Lewis,*[b] *and David K. Smith **[a]

[a] Department of Chemistry, University of York, Heslington, York, YO10 5DD, UK.
Email: David.smith@york.ac.uk
[b] Kilfrost Ltd, Albion Works, Haltwhistle, NE49 0HJ, UK

SUPPLEMENTARY INFORMATION

S1 Characterisation of Gelators
S2 Gel Analysis
S3 Hansen Solubility Parameters (HSPs)
S4 Rheology
S5 Electron Microscopy
S6 Water Spray Endurance Test (WSET)
S7 Aerodynamic Testing Using Rheology
S8 NMR Spectra of Key Gelators
S9 References

S1 Characterisation of Gelators

S1.1 1,3:2,4-Di-*O*-(4'-methoxybenzylidene)–D–Sorbitol (DBS-OCH_3).[1] General procedure A was used with 4-methoxybenzaldehyde. DBS-OCH_3 was obtained as a white powder (5.50 g, 13.15 mmol, 52% yield). ^{1}H NMR (400 MHz, DMSO-d_6) δ: 7.40-7.36 (m, 4H, Ar-**H**), 6.94-6.90 (m, 4H, Ar-**H**), 5.59 (s, 2H, Ar-C**H**), 4.42-4.39 (m, 1H, C**H**-sugar), 4.17-4.10 (m, 3H, C**H$_2$**-sugar, C**H**-sugar), 3.89-3.86 (m, 1H, CH_2O**H**), 3.80 (dd, J = 9.2, 1.6 Hz, 1H, C**H**-sugar), 3.75 (s, 6H, OC**H**$_3$), 3.60–3.55 (m, 2H, C**H$_2$**-sugar), 3.44–3.38 (m, 1H, C**H**-sugar). ^{13}C NMR (100 MHz, DMSO-d_6) δ: 159.93, 159.88, 131.72, 131.45, 128.02, 127.99, 113.80, 113.74 (all Ar-**C**), 99.83, 99.76 (both Ar-**C**H), 78.15, 70.51 (both **C**H-sugar), 69.80 (**C**H_2-sugar), 68.84, 68.24 (both **C**H-sugar), 63.14 (**C**H_2-sugar), 55.63 (O-**C**H_3). ATR-FTIR ν_{max} (cm^{-1}): 3210 (O-H), 2928 (C-H), 1588 (C=C), 1094 (C-O), 1005 (C-O). ESI-MS: Calculated for ($C_{22}H_{26}O_8$) $[M+Na]^+$ *m/z* = 441.1518. Found $[M+Na]^+$ *m/z* = 441.1520 (100%). Melting Point: 126–130 °C.

S1.2 1,3:2,4-Di-*O*-(4'-(methylthio)benzylidene)–D–Sorbitol (DBS-SCH_3). General procedure A was used with 4-methylthiobenzaldehyde. DBS-SCH_3 was obtained as a white powder (9.95 g, 22.10 mmol, 88% yield). ^{1}H NMR (400 MHz, DMSO-d_6) δ: 7.42-7.38 (m, 4H, Ar-**H**), 7.28-7.25 (m, 4H, Ar-**H**), 5.62 (s, 2H, Ar-C**H**), 4.20–4.11 (m, 3H, CHO**H** and C**H**$_2$-sugar), 3.94-3.91 (m, 1H, C**H**-sugar), 3.84-3.82 (m, 1H, CH_2O**H**-sugar), 3.77–3.74 (m, 2H, C**H**-sugar x2), 3.61-3.58 (m, 2H, C**H**$_2$-sugar), 3.46-3.41 (m, 1H, C**H**-sugar), 2.47 (s, 6H, SC**H**$_3$). ^{13}C NMR (100 MHz, DMSO-d_6) δ: 138.57, 138.46, 135.35, 135.08, 126.69, 126.66, 125.32, 125.26 (all Ar-**C**), 98.95, 98.90 (both Ar-**C**H), 77.50, 69.95 (both **C**H-sugar), 69.20 (**C**H_2-sugar), 68.27, 67.62 (both **C**H-sugar), 62.52 (**C**H_2-sugar), 14.60 (S**C**H_3). ATR-FTIR ν_{max} (cm^{-1}): 3220 (OH), 2916 (CH), 1497 (C=C), 1094 (C-O), 733 (C-S). ESI-MS: Calculated for ($C_{22}H_{26}O_6S_2$) $[M+Na]^+$ *m/z* = 473.1069. Found $[M+Na]^+$ *m/z* = 473.1063 (100%). Melting point: 140-143 °C.

S1.3 1,3:2,4-Di-*O*-(4'-(trifluoromethyl)benzylidene)–D–Sorbitol (DBS-CF_3). General procedure A was used with 4-trifluoromethylbenzaldehyde. DBS-CF_3 was obtained as a white powder (3.69 g, 7.47 mmol, 50% yield). ^{1}H NMR (400 MHz, DMSO-d_6) δ: 7.79–7.65 (m, 8H, Ar-**H**), 5.78 (s, 2H, Ar-C**H**), 4.40 (d, J = 5.9 Hz, 1H, CHO**H**), 4.30–4.16 (m, 2H, C**H**$_2$-sugar), 4.13-4.07 (m, 1H, C**H**-sugar), 4.02 (s, 1H, CH_2O**H**), 3.91 (dd, J = 9.4, 1.6 Hz, 1H, C**H**-sugar), 3.63-3.60 (m, 1H, C**H**-sugar), 3.58-3.54 (m, 2H, C**H**$_2$-sugar), 3.49-3.45 (m, 1H, C**H**-sugar). ^{13}C NMR (100 MHz, DMSO-d_6) δ: 142.78, 142.53, 137.81, 128.10, 127.44, 127.31, 127.00, 126.98 (all Ar-**C**), 125.02 (**C**F_3), 98.24, 98.16 (both Ar-**C**H), 77.48, 70.07 (both **C**H-sugar), 69.27 (**C**H_2-sugar), 68.43, 67.61 (both **C**H-sugar), 62.50 (**C**H_2-sugar). ATR-FTIR ν_{max} (cm^{-1}): 3253 (OH), 2938 (CH), 1522 (C=C), 1094 (C-O), 1322 (C-F). ESI-MS: Calculated for ($C_{22}H_{20}F_6O_6$) $[M+Na]^+$ *m/z* = 517.070. Found $[M+Na]^+$ *m/z* = 517.1056 (100%). Melting point:156-160 °C.

S1.4 1,3:2,4-Di-*O*-(4'-nitrobenzylidene)–D–Sorbitol (DBS-NO_2).[2] General procedure A was used with 4-nitrobenzaldehyde. DBS-NO_2 was obtained as a yellow powder (1.05 g, 2.34 mmol, 18% yield). ^{1}H NMR (400 MHz, DMSO-d_6) δ: 8.28-8.25 (m, 4H, Ar-**H**), 7.76-7.71 (m, 4H, Ar-**H**), 5.83 (s, 2H, Ar-C**H**), 4.98 (d, J = 5.9 Hz, 1H, CHO**H**), 4.51-4.49 (m, 1H, C**H**-sugar), 4.31–4.19 (m, 2H, C**H**$_2$-sugar), 4.06 (s, 1H, CH2O**H**), 3.94-3.92 (m, 1H, C**H**-sugar), 3.78 (dd, J = 6.0, 2.6 Hz, 1H, C**H**-sugar), 3.64-3.59 (m, 2H, C**H**$_2$-sugar), 3.50-3.44 (m, 1H, C**H**-sugar). ^{13}C NMR (100 MHz, DMSO-d_6) δ: 147.68,

147.65, 145.17, 144.91, 127.60, 127.59, 123.46, 123.37 (all Ar-**C**), 97.97, 97.88 (both Ar-**C**H), 77.51, 70.17 (both **C**H-sugar), 69.34 (**C**H_2-sugar), 68.54, 67.59 (both **C**H-sugar), 62.52 (**C**H_2-sugar). ATR-FTIR ν_{max} (cm^{-1}): 3367 (OH), 2876 (CH), 1520 (C=C/N=O), 1454, 1339 (N=O), 1093 (C-O). ESI-MS: Calculated for ($C_{20}H_{20}N_2O_{10}$) $[M+Na]^+$ *m/z* 471.1012. Found $[M+Na]^+$ *m/z* = 471.1010 (100%). Melting point: 173-187 °C. Characterisation data were in agreement with reference 2.

S1.5 1,3:2,4-Di-*O*-(4'-ethoxybenzylidene)–D–Sorbitol (DBS-OCH_2CH_3). General procedure A was used with 4-ethoxybenzaldehyde. DBS-OCH_2CH_3 was obtained as a white powder (6.54 g, 14.66 mmol, 58% yield). ^{1}H NMR (400 MHz, DMSO-d_6) δ: 7.38-7.34 (m, 4H, Ar-**H**), 6.92-6.90 (m, 4H, Ar-**H**), 5.58 (s, 2H, Ar-C**H**), 4.13-4.09 (m, 1H, CHO**H**), 4.04-3.99 (m, 5H, OC**H**$_2$CH$_3$ and C**H**-sugar), 3.88 (s, 1H, CH$_2$O**H**), 3.81-3.79 (m, 2H, C**H**$_2$-sugar), 3.76–3.71 (m, 1H, C**H**-sugar), 3.60–3.55 (m, 2H, C**H**$_2$-sugar), 3.44–3.35 (m, 2H, C**H**-sugar), 1.32 (t, J = 7.0 Hz, 6H, OCH$_2$C**H**$_3$). ^{13}C NMR (100 MHz, DMSO-d_6) δ: 159.21, 159.16, 131.58, 131.32, 128.02, 127.99, 114.25, 114.19, (all Ar-**C**), 99.86, 99.80 (both Ar-**C**H), 78.16, 70.54 (both **C**H-sugar), 69.83 (**C**H_2-sugar), 68.86, 68.27 (both **C**H-sugar), 63.54 (**C**H_2-sugar), 63.20 (CH$_3$**C**H$_2$O) 15.15 (**C**H$_3$CH$_2$O). ATR-FTIR ν_{max} (cm^{-1}): 3232 (OH), 2932 (CH), 1585 (C=C), 1088 (C-O), 1011 (C-O). ESI-MS: Calculated for ($C_{24}H_{30}O_8$) $[M+Na]^+$ *m/z* = 469.1820. Found $[M+Na]^+$ *m/z* = 469.1833 (100%). Melting Point: 123-126 °C

S1.6 1,3:2,4-Di-*O*-(4'-(methylsulfonyl)benzylidene)–D–Sorbitol (DBS-SO_2CH_3). General procedure A was used with 4-methylsulfonylbenzaldehyde. DBS-SO_2CH_3 was obtained as a yellow powder (5.03 g, 9.78 mmol, 65% yield). ^{1}H NMR (400 MHz, DMSO-d_6) δ: 7.94-7.91 (m, 4H, Ar-**H**), 7.72-7.69 (m, 4H, Ar-**H**), 5.77 (s, 2H, Ar-C**H**), 4.24 (d, J = 2.0Hz, 1H, CHO**H**), 4.22–4.14 (m, 3H, C**H**$_2$-sugar and C**H**-sugar), 4.00 (d, J = 1.7 Hz, 1H, CH$_2$O**H**), 3.88 (dd, J = 9.4, 1.6 Hz, 1H, C**H**-sugar), 3.76-3.72 (m, 1H C**H**-sugar), 3.60–3.54 (m, 2H, C**H**$_2$-sugar), 3.46-3.41 (m, 1H, C**H**-sugar), 3.16-3.13 (m, 6H, SO$_2$C**H**$_3$). ^{13}C NMR (100 MHz, DMSO-d_6) δ: 143.70, 143.45, 140.95, 140.89, 127.18, 127.16, 126.95, 126.86, (all Ar-**C**), 98.18, 98.09 (both Ar-**C**H), 77.47, 70.06 (both **C**H-sugar), 69.27 (**C**H_2-sugar), 68.42, 67.61 (**C**H-sugar), 62.51 (**C**H_2-sugar), 43.57, 43.55 (both SO$_2$**C**H$_3$). ATR-FTIR ν_{max}(cm^{-1}): 3269 (OH), 2929 (CH), 1519 (C=C), 1293 (S=O), 1093 (C-O). ESI-MS: Calculated for ($C_{22}H_{26}O_{10}S_2$) $[M+Na]^+$ *m/z* = 537.0863. Found $[M+Na]^+$ *m/z* = 537.0860 (100%). Melting Point: 149-152 °C.

S2 Gel Analysis

Table S1. Aliquots of solvent required to make gels with DF+.

% MPG in Sample	Volume of DF+ added (mL)	Volume of Water added (mL)
**80%**	1	0
**72%**	0.9	0.1
**64%**	0.8	0.2
**56%**	0.7	0.3
**48%**	0.6	0.4
**40%**	0.5	0.5
**32%**	0.4	0.6
**24%**	0.3	0.7
**16%**	0.2	0.8
**8%**	0.1	0.9

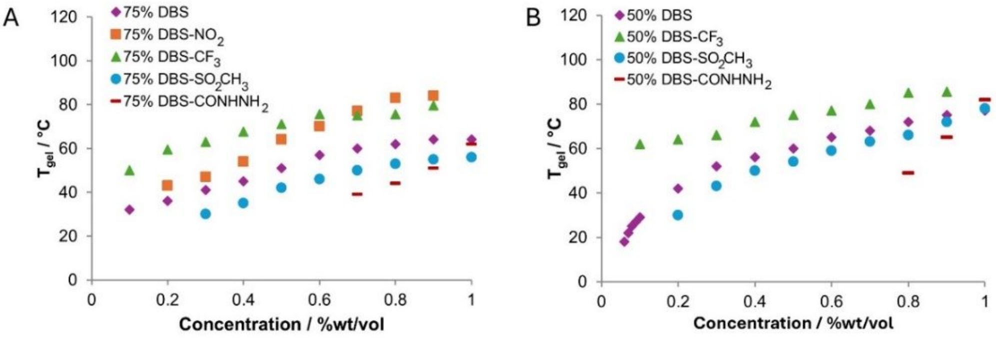

Figure S1. Concentration dependent T_{gel} values in (A) 75:25 MPG:H_2O and (B) 50:50 MPG:H_2O for different LMWGs (purple diamonds = DBS, orange squares = DBS-NO_2, green triangles = DBS-CF_3, blue circles = DBS-SO_2CH_3, red bars = DBS-$CONHNH_2$).

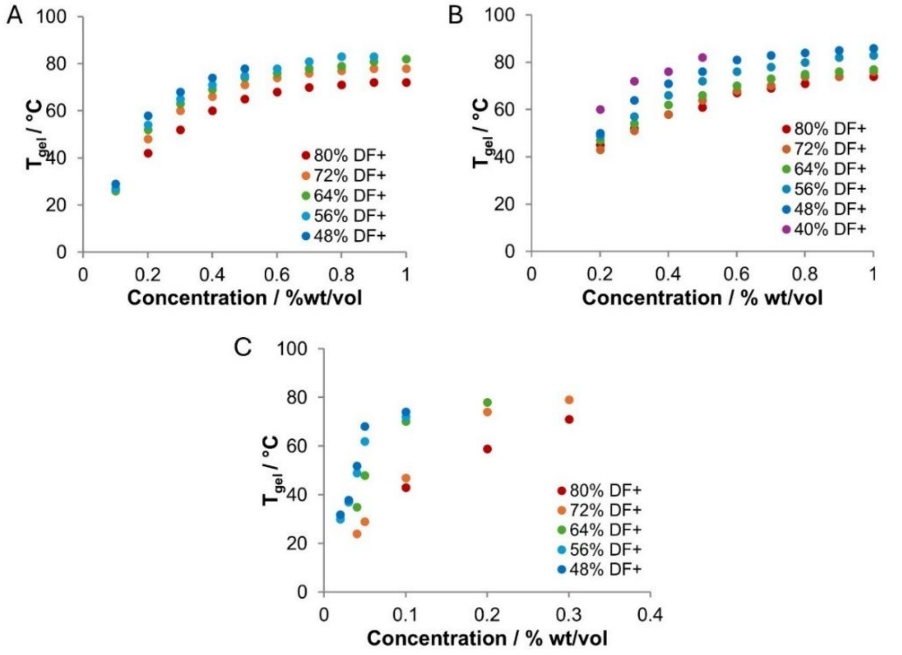

Figure S2. Concentration dependent T_{gel} values for (A) DBS, (B) DBS-OCH_3 and, (C) DBS-SCH_3 in DF+ at different dilutions (red = 80% MPG, orange = 72% MPG, green = 64% MPG, pale blue = 56% MPG, dark blue = 48% MPG, purple = 40% MPG).

Table S2. Aliquots of solvent required to make scaled-up gels with DF+.

% MPG in Sample	Volume of DF+ added (mL)	Volume of Water added (mL)
**80%**	200	0
**72%**	180	20
**64%**	160	40
**56%**	140	60
**48%**	120	80
**40%**	100	100
**32%**	80	120
**24%**	60	140
**16%**	40	160

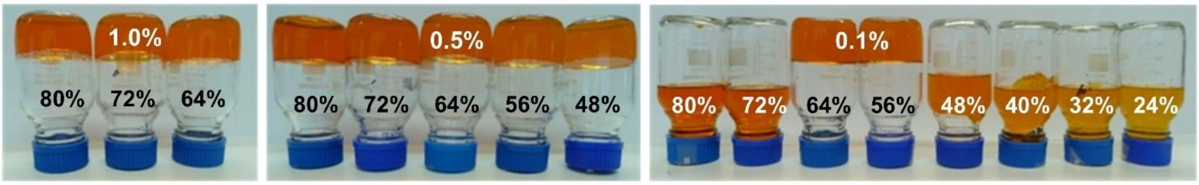

Figure S3. Scaled-up samples of DBS gels at high (1.0% wt/vol), medium (0.5% wt/vol) and low (0.1% wt/vol) concentration in DF+ and dilutions of DF+ using vial inversion to indicate the presence or absence of a gel.

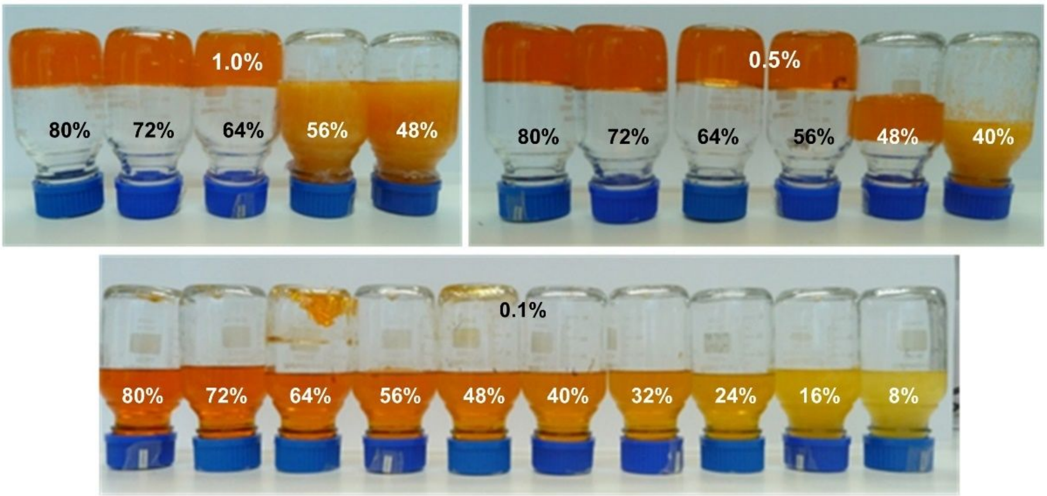

Figure S4. Scaled-up samples of DBS-OCH_3 gels at high (1.0% wt/vol), medium (0.5% wt/vol) and low (0.1% wt/vol) concentration in DF+ and dilutions of DF+ using vial inversion to indicate the presence or absence of a gel.

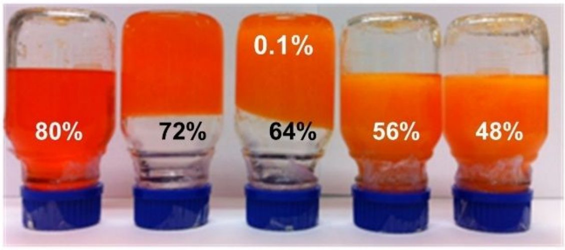

Figure S5. Scaled-up samples of DBS-SCH_3 gels at low (0.1% wt/vol) concentration in DF+ and dilutions of DF+ using vial inversion to indicate the presence or absence of a gel.

S3 Hansen Solubility Parameters (HSPs)

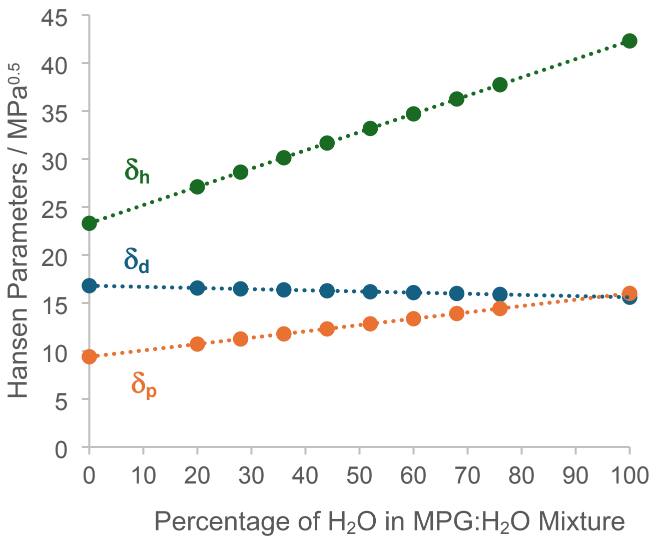

Figure S6. Hansen Solvent Parameters (HSPs) for mixtures of MPG and H_2O: δ_d = dispersion parameter, δ_p = polar parameter, δ_h = hydrogen bonding parameter.

S4 Rheology

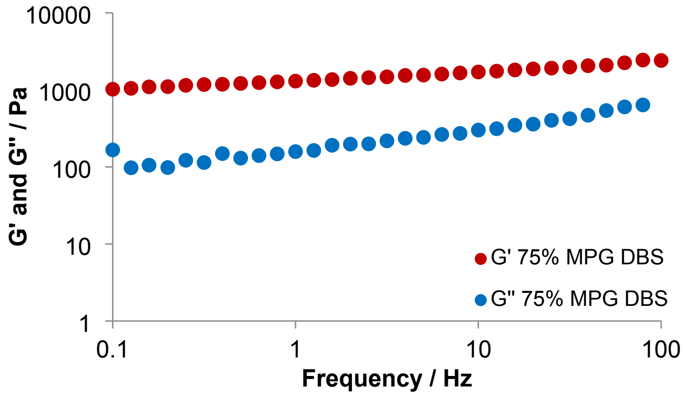

Figure S7. Frequency sweep for DBS (0.5% wt/vol) in MPG:H_2O 75:25 indicating gel-like behaviour (G'>G") and invariance of G' (red) and G" (blue) with frequency.

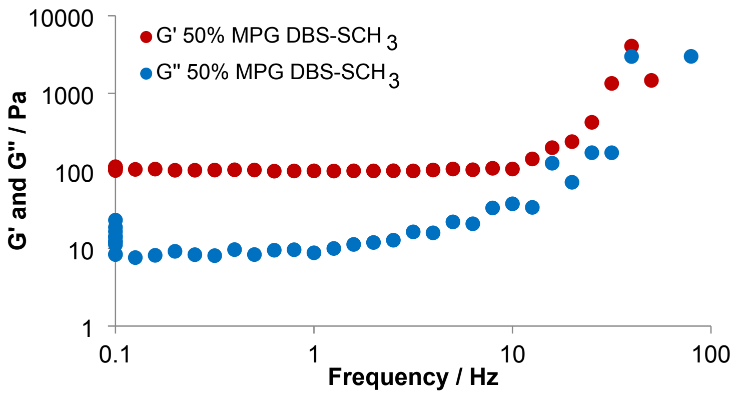

Figure S8. Frequency sweep for DBS-SCH_3 (0.1% wt/vol) in MPG:H_2O 50:50 indicating gel-like behaviour (G'>G") and invariance of G' (red) and G" (blue) with frequency below 10 Hz.

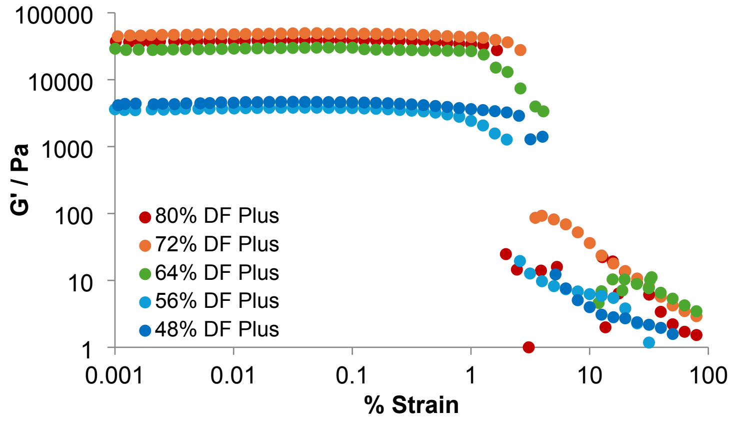

Figure S9. Amplitude sweep for DBS (0.5% wt/vol) in DF+ showing G' values at a range of dilutions (red = 80% MPG, orange = 72% MPG, green = 64% MPG, pale blue = 56% MPG, dark blue = 48% MPG).

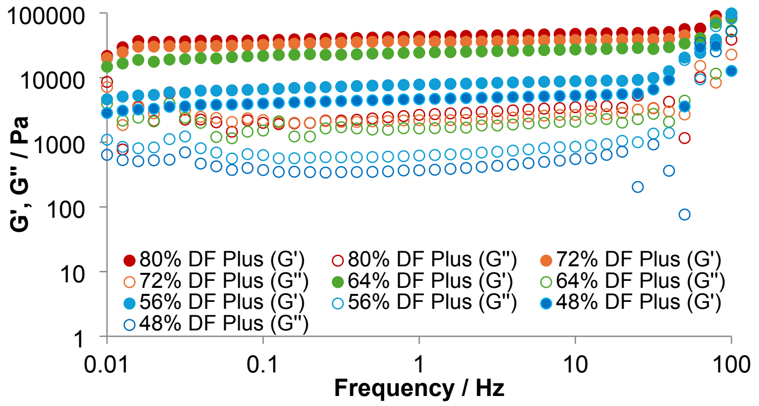

Figure S10. Frequency sweep for DBS (0.5% wt/vol) in DF+ showing G' (filled circles) and G" (open circles) at a range of dilutions (red = 80% MPG, orange = 72% MPG, green = 64% MPG, pale blue = 56% MPG, dark blue = 48% MPG).

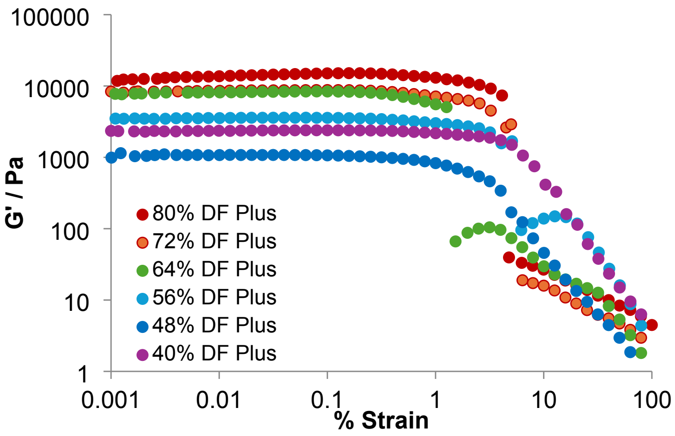

Figure S11. Amplitude sweep for DBS-OCH$_3$ (0.5% wt/vol) in DF+ showing G' values at a range of dilutions (red = 80% MPG, orange = 72% MPG, green = 64% MPG, pale blue = 56% MPG, dark blue = 48% MPG, purple = 40% MPG).

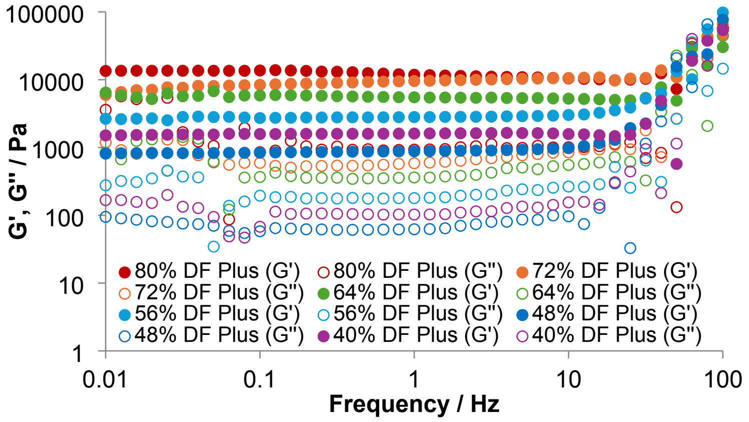

Figure S12. Frequency sweep for DBS-OCH$_3$ (0.5% wt/vol) in DF+ showing G' (filled circles) and G" (open circles) at a range of dilutions (red = 80% MPG, orange = 72% MPG, green = 64% MPG, pale blue = 56% MPG, dark blue = 48% MPG, purple = 40% MPG).

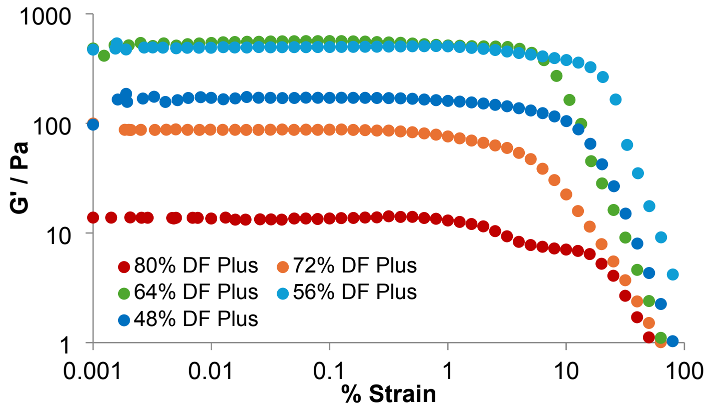

Figure S13. Amplitude sweep for DBS-SCH$_3$ (0.1% wt/vol) in DF+ showing G' values at a range of dilutions (red = 80% MPG, orange = 72% MPG, green = 64% MPG, pale blue = 56% MPG, dark blue = 48% MPG).

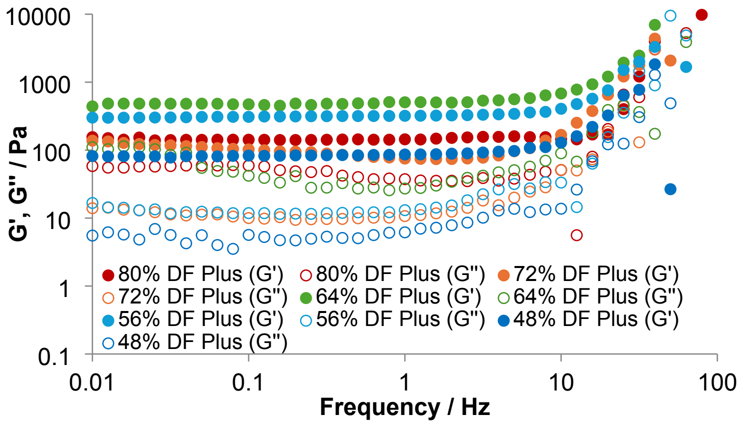

Figure S14. Frequency sweep for DBS-SCH_3 (0.1% wt/vol) in DF+ showing G' (filled circles) and G" (open circles) at a range of dilutions (red = 80% MPG, orange = 72% MPG, green = 64% MPG, pale blue = 56% MPG, dark blue = 48% MPG).

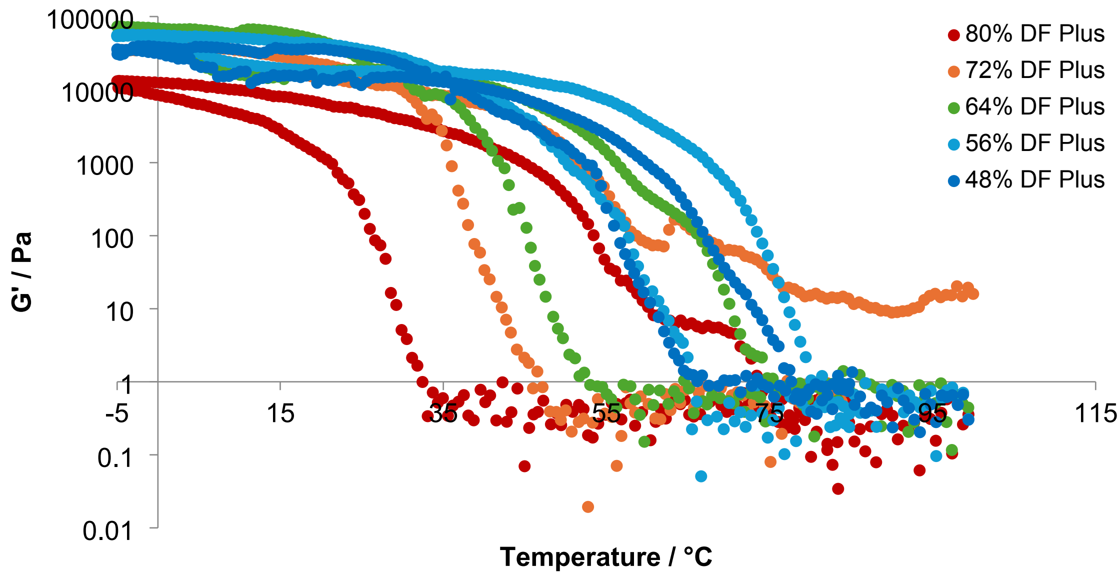

Figure S15. Temperature sweep for DBS (0.5% wt/vol) in DF+ showing G' at a range of dilutions (red = 80% MPG, orange = 72% MPG, green = 64% MPG, pale blue = 56% MPG, dark blue = 48% MPG).

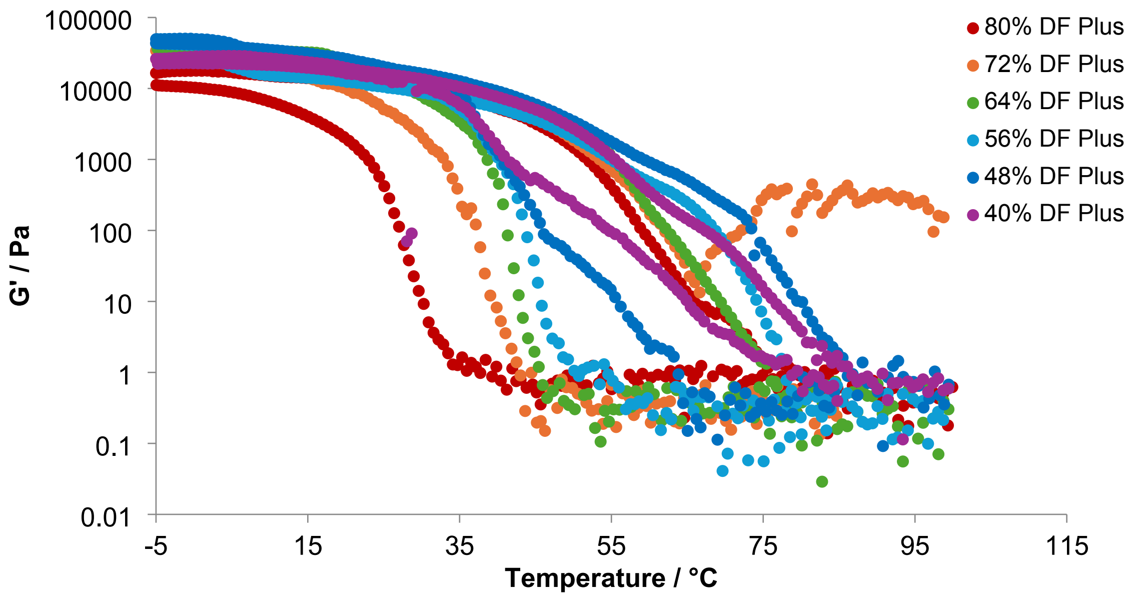

Figure S16. Temperature sweep for DBS-OCH_3 (0.5% wt/vol) in DF+ showing G' at a range of dilutions (red = 80% MPG, orange = 72% MPG, green = 64% MPG, pale blue = 56% MPG, dark blue = 48% MPG, purple = 40% MPG).

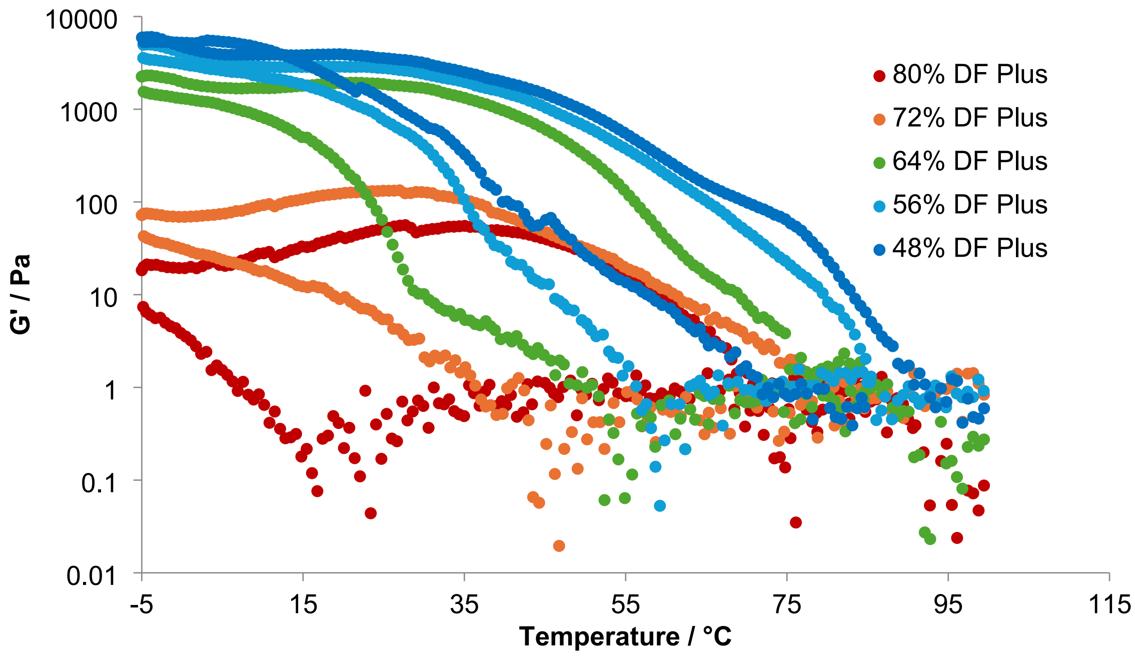

Figure S17. Temperature sweep for DBS-SCH_3 (0.1% wt/vol) in DF+ showing G' at a range of dilutions (red = 80% MPG, orange = 72% MPG, green = 64% MPG, pale blue = 56% MPG, dark blue = 48% MPG).

Table S3. Gel formation (T_f) and gel dissolution (T_d) temperatures for DBS and DBS-OCH$_3$ (both 0.5% wt/vol) and DBS-SCH$_3$ (0.1% wt/vol) in DF Plus and DF Plus dilutions (*I* = Insoluble).

**Solvent (% MPG)**	**DBS (0.5% wt/vol)**		**DBS-OCH$_3$ (0.5% wt/vol)**		**DBS-SCH$_3$ (0.1% wt/vol)**	
	onset-T_f (°C)	T_d (°C)	onset-T_f (°C)	T_d (°C)	onset-T_f (°C)	T_d (°C)
**80**	31.8	73.5	32.9	62.1	9.5	67.9
**72**	46.5	61.4	42.5	68.1	32.0	72.8
**64**	51.2	72.1	44.9	71.4	44.1	76.7
**56**	65.0	79.5	48.6	76.1	54.1	84.1
**48**	63.9	76.1	61.6	82.1	68.6	88.1
**40**	*I*	*I*	71.9	86.8	*I*	*I*

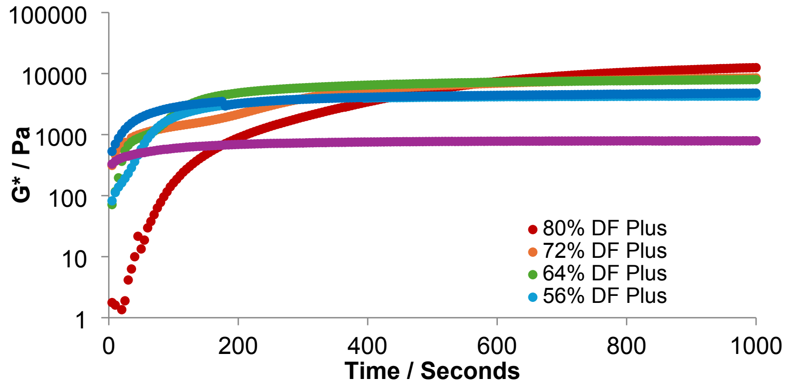

Figure S18. Time-resolved rheology for DBS-OCH$_3$ (0.5% wt/vol) in DF+ showing the evolution of G* over time at a range of dilutions (red = 80% MPG, orange = 72% MPG, green = 64% MPG, pale blue = 56% MPG, dark blue = 48% MPG, purple = 40% MPG).

S5 Electron Microscopy

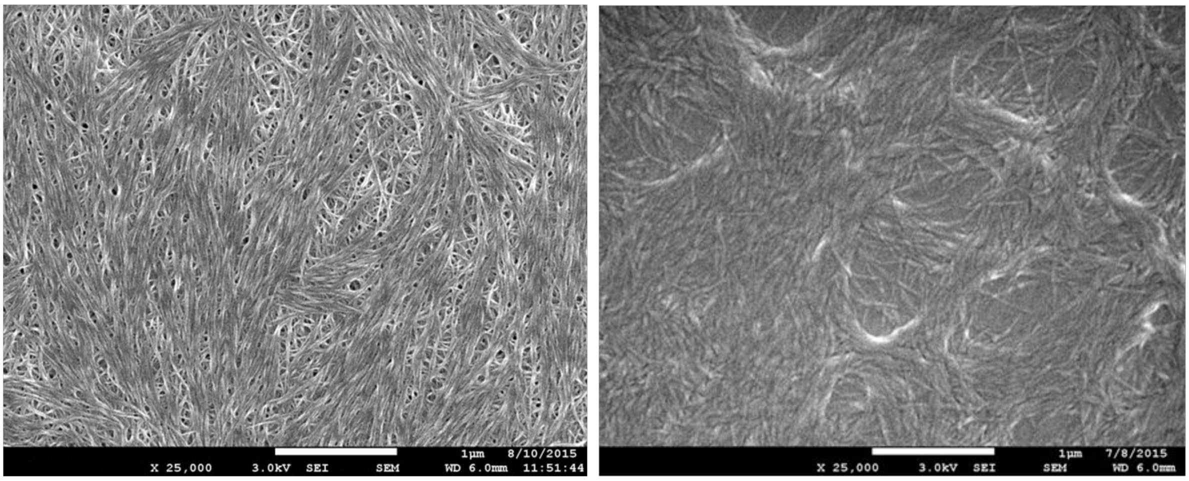

Figure S19. SEM images of DBS, in 75:25 MPG:H_2O when gel formation is performed using (left) fast cooling conditions and (right) slow cooling conditions. All scale bars = 1 μm.

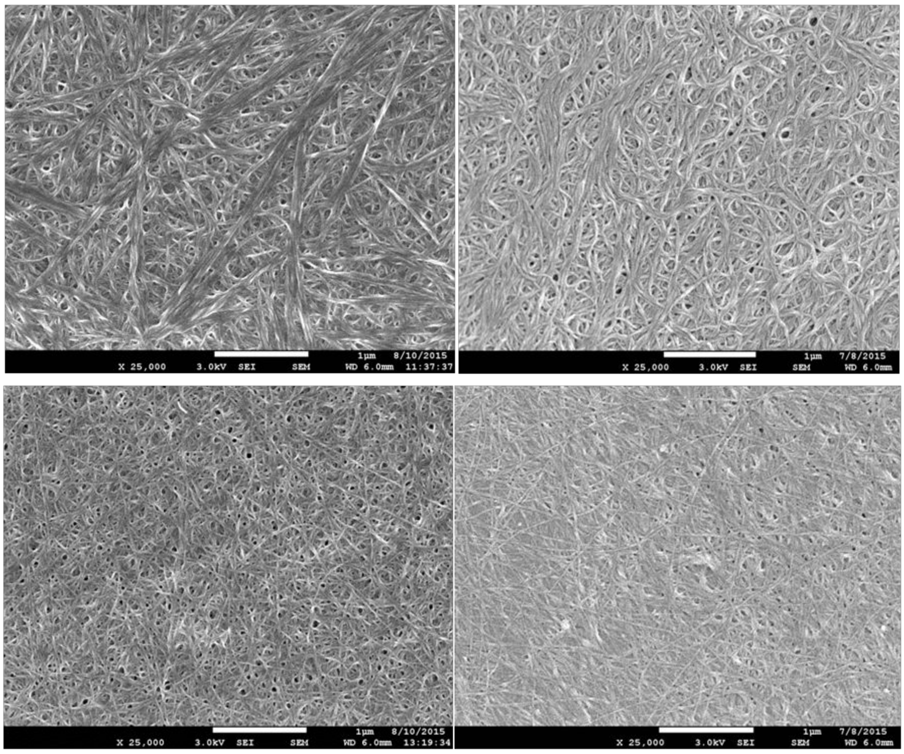

Figure S20. SEM images of DBS-OCH_3, in (top) 100% MPG and (bottom) 75:25 MPG:H_2O when gel formation is performed using (left) fast cooling conditions and (right) slow cooling conditions. All scale bars = 1 μm.

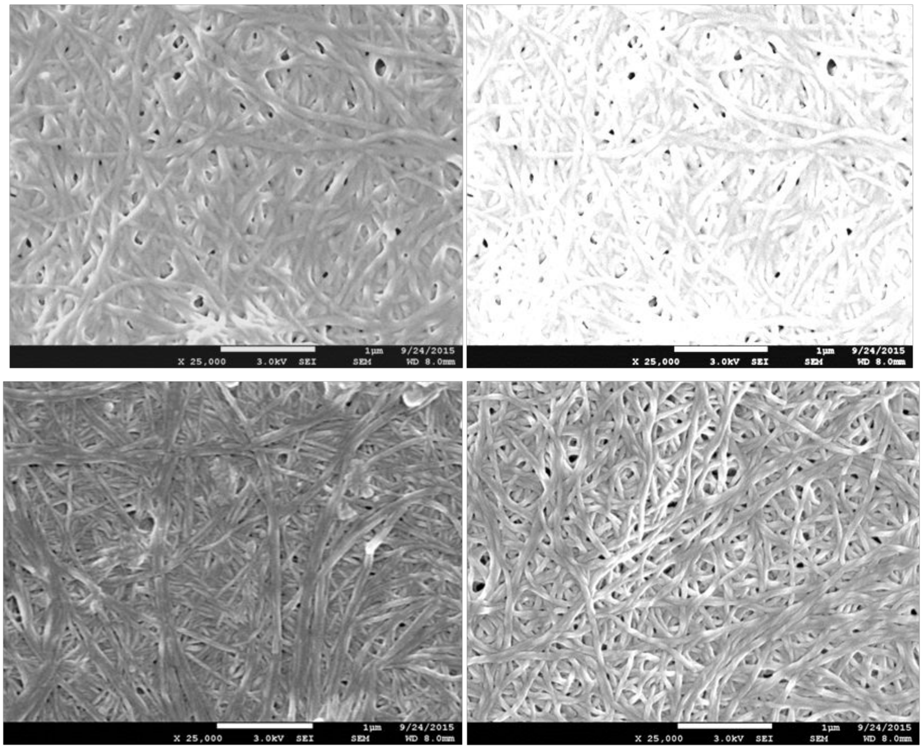

Figure S21. SEM images of DBS-SCH_3, in (top) 100% MPG and (bottom) 75:25 MPG:H_2O when gel formation is performed using (left) fast cooling conditions and (right) slow cooling conditions. All scale bars = 1 μm.

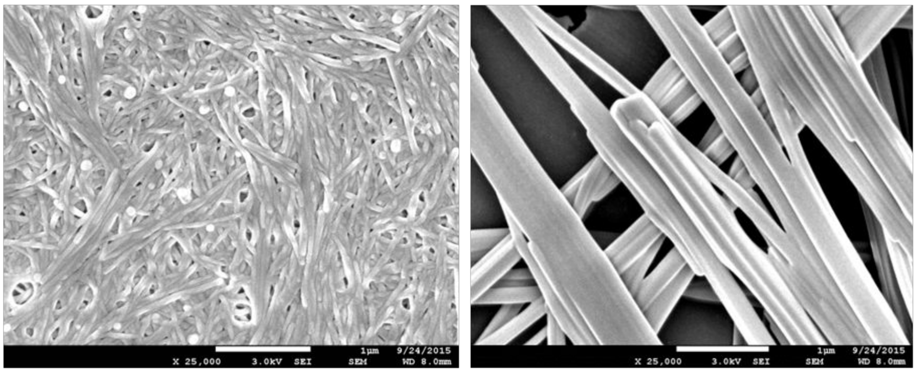

Figure S22. Additional SEM images of DBS-SCH_3, in (50:50 MPG:H_2O when gel formation is performed using (left) fast cooling conditions and (right) slow cooling conditions showing the impact of gel fabrication method on nanoscale morphology. All scale bars = 1 μm.

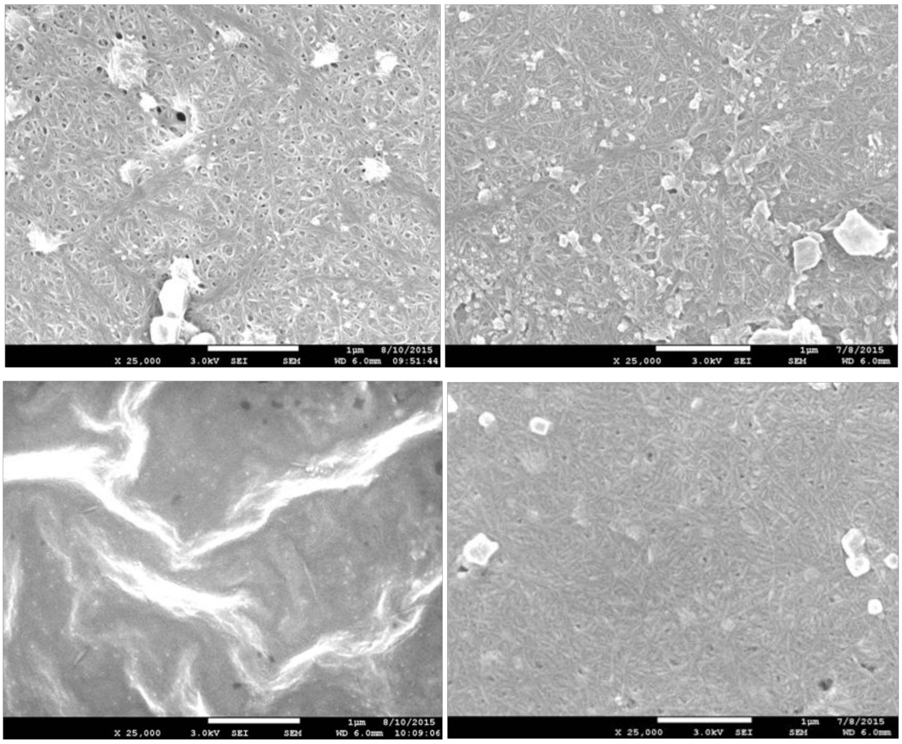

Figure S23. SEM images of DBS, in (top) DF+ (80% MPG) and (bottom) diluted DF+ (48% MPG) when gel formation is performed using (left) fast cooling conditions and (right) slow cooling conditions. All scale bars = 1 μm.

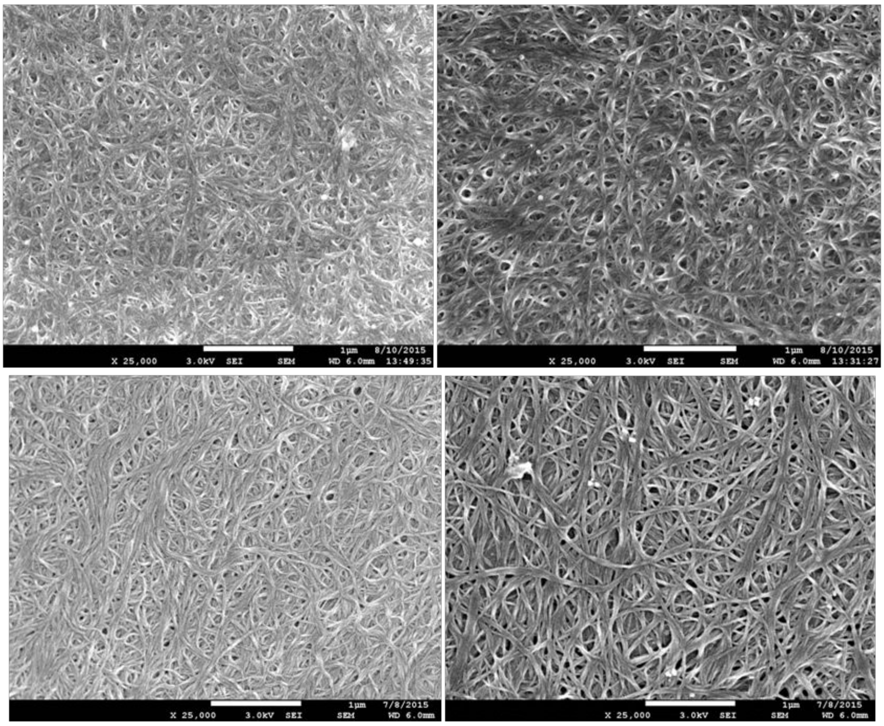

Figure S24. SEM images of DBS-OCH_3, in (top) DF+ (80% MPG) and (bottom) diluted DF+ (40% MPG) when gel formation is performed using (left) fast cooling conditions and (right) slow cooling conditions. All scale bars = 1 μm.

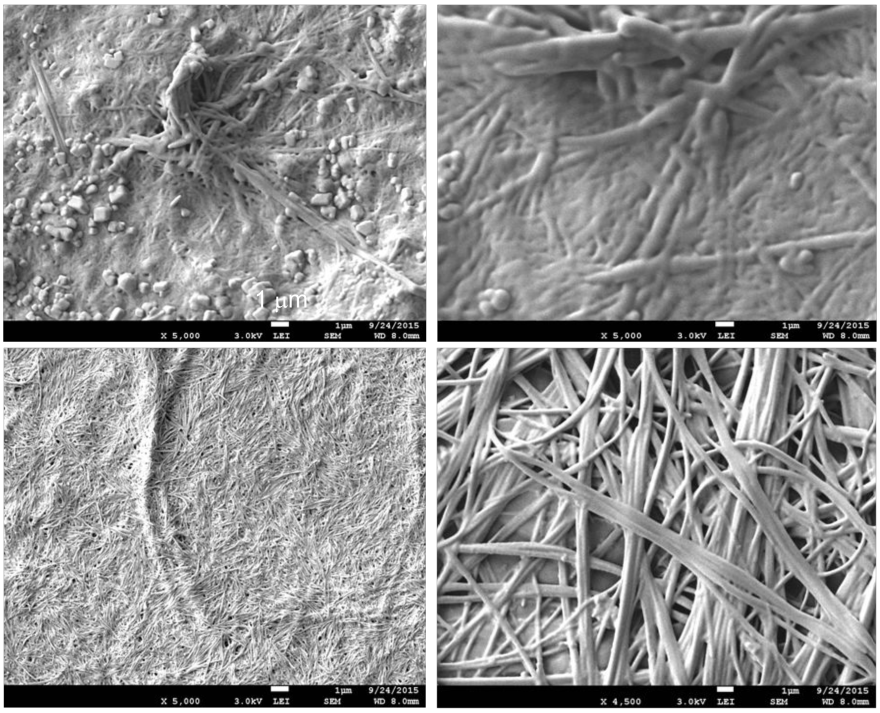

Figure S25. SEM images of DBS-SCH_3, in (top) DF+ (80% MPG) and (bottom) diluted DF+ (48% MPG) when gel formation is performed using (left) fast cooling conditions and (right) slow cooling conditions. All scale bars = 1 μm.

S6 Water Spray Endurance Test (WSET)

Table S4. Minimum WSET holdover times as specified by the relevant regulator.

Dilution	Minimum WSET Holdover Time (minutes)		
	Type II	Type III	Type IV
**100%**	30	20	80
**75%**	20	Report	20
**50%**	5	Report	5

Table S5. Holdover times for Type I DF+ and diluted samples DF Plus dilutions.

% MPG in DF+	Holdover Time (min.sec)	Type I (Pass/Fail)
**80**	7.07±0.16	Pass
**72**	6.35±0.16	Pass
**64**	6.13±0.13	Pass
**56**	5.51±0.13	Pass
**48**	5.41±0.13	Pass
**40**	4.37±0.10	Pass
**32**	3.15±0.07	Pass

S7 Aerodynamic Testing using Rheology

Table S6. Percentage of G' value remaining after increasing strain on DBS (0.5% wt/vol) and DBS-OCH_3 (0.5% wt/vol) and DBS-SCH_3 (0.1% wt/vol) in DF+ (80% MPG).

% Strain	% G' Remaining after increasing strain		
	DBS (0.5% wt/vol)	DBS-OCH_3 (0.5% wt/vol)	DBS-SCH_3 (0.1% wt/vol)
**10**	0.53	1.19	1.30
**50**	0.15	0.15	0.17
**100**	0.09	0.01	0.18

Table S7. Percentage recovery of G' value after removing strain on DBS (0.5% wt/vol) and DBS-OCH_3 (0.5% wt/vol) and DBS-SCH_3 (0.1% wt/vol) in DF+ (80% MPG).

% Strain	% Recovery of G' value after removal of strain		
	DBS (0.5% wt/vol)	DBS-OCH_3 (0.5% wt/vol)	DBS-SCH_3 (0.1% wt/vol)
**10**	42	59	84
**50**	25	49	80
**100**	7	14	31

S8 NMR Spectra of Key gelators

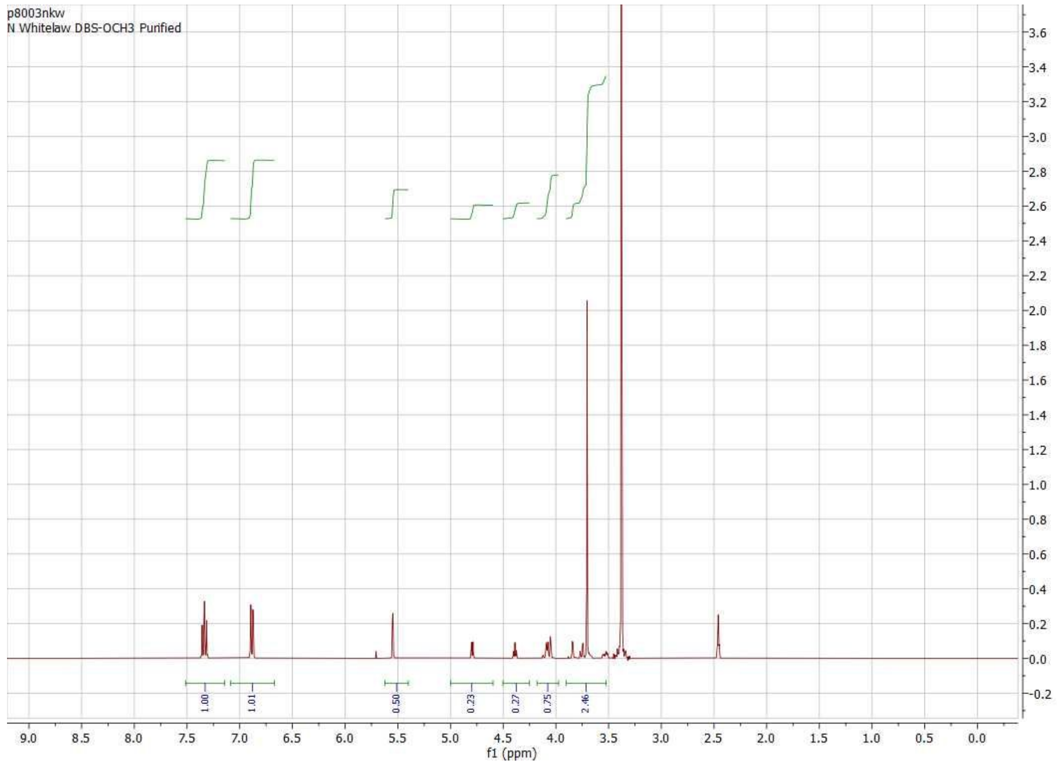

Figure S26. ^{1}H NMR spectrum of DBS-OCH_3 in d_6-DMSO.

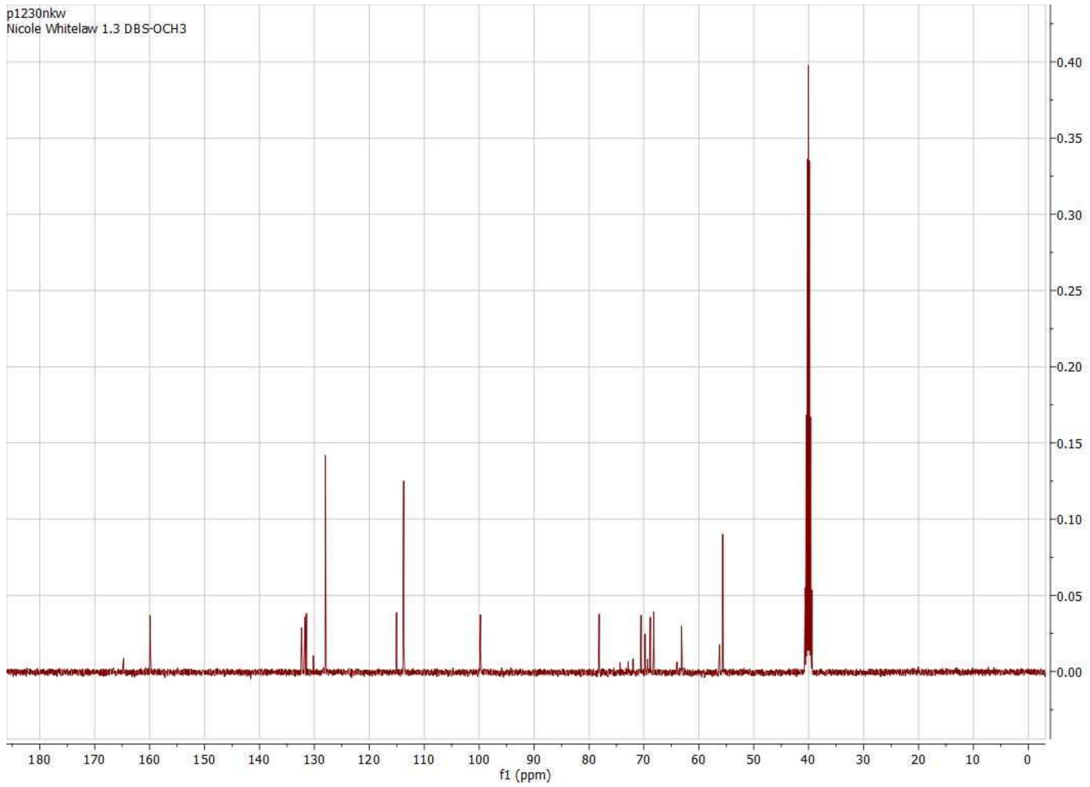

Figure S27. ^{13}C NMR spectrum of DBS-OCH_3 in d_6-DMSO.

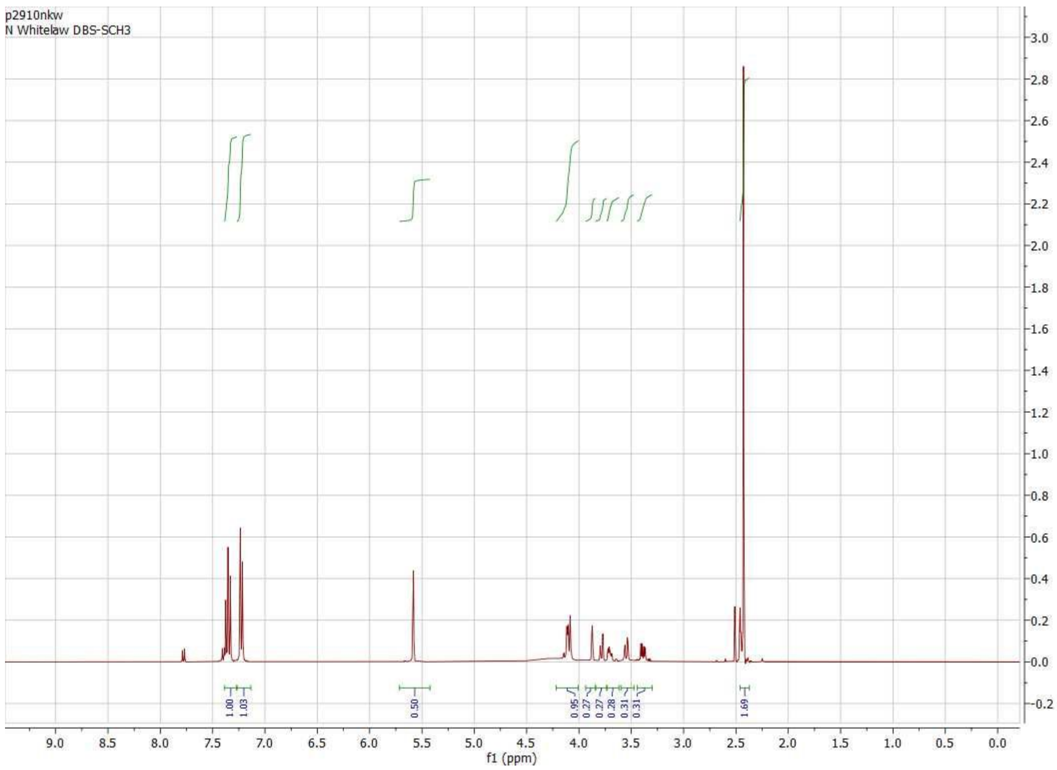

Figure S28. ^{1}H NMR spectrum of DBS-SCH_3 in d_6-DMSO.

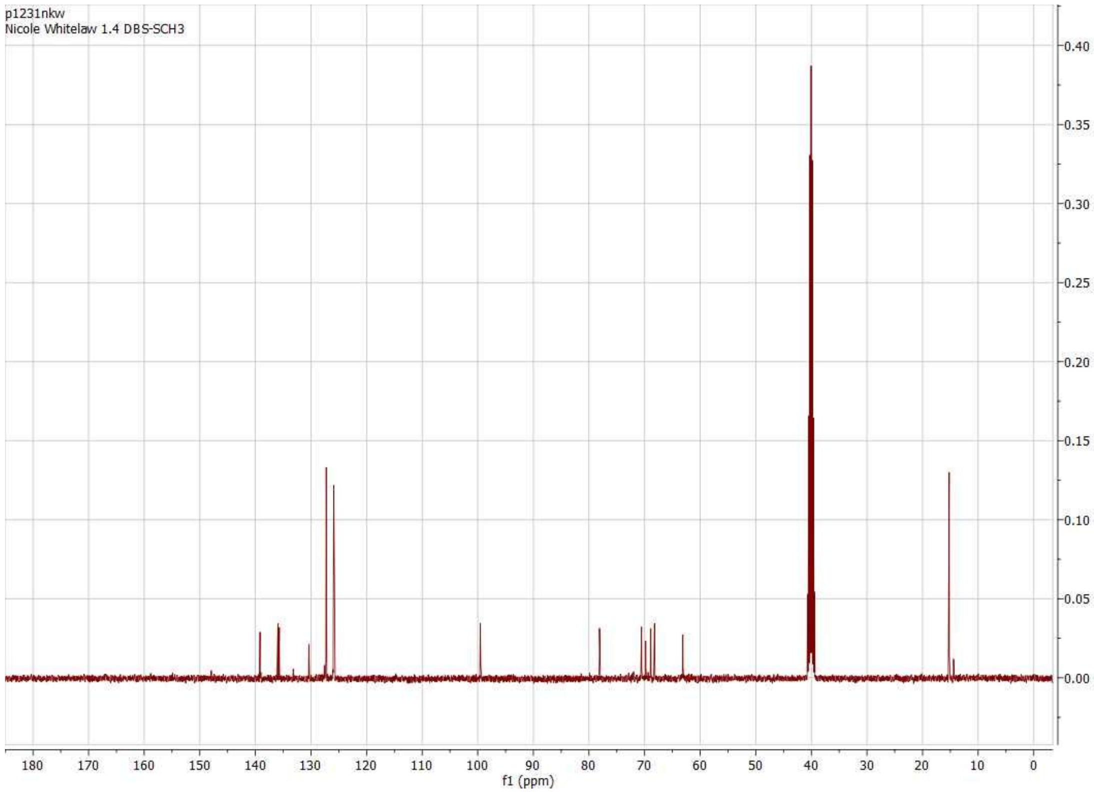

Figure S29. ^{13}C NMR spectrum of DBS-SCH_3 in d_6-DMSO.

S9 References

1. Stan, R.; Ott, C.; Sulca, N.; Lungu, A.; Iovu, H. Functionalized D-Sorbitol-Based Organogelators for Dental Materials (I), *Materiale Plastice*, **2009**, *46*, 230-235.
2. Stan, R. Rosca, S.; Ott, C.; Rosca, S.; Perez, E.; Rico-Lattes, I.; Lattes, A. D-Sorbitol Based Organogelators with Nitrogen Groups. *Rev. Roum. Chim.*, **2006**, *51*, 609–613.
